# Synthesis of chiral *N*-phosphinyl α-imino esters and their application in asymmetric synthesis of α-amino esters by reduction

**DOI:** 10.3762/bjoc.10.57

**Published:** 2014-03-13

**Authors:** Yiwen Xiong, Haibo Mei, Lingmin Wu, Jianlin Han, Yi Pan, Guigen Li

**Affiliations:** 1 School of Chemistry and Chemical Engineering, State of Key Laboratory of Coordination, Nanjing University, Nanjing, 210093, China; 2 Institute for Chemistry & BioMedical Sciences, Nanjing University, Nanjing, 210093, China; 3 High-Tech Research Institute of Nanjing University, Changzhou, 213164, China; 4 Department of Chemistry and Biochemistry, Texas Tech University, Lubbock, Texas, 79409-1061, USA

**Keywords:** α-amino ester, α-imino ester, group-assisted purification, phosphinyl auxiliary, reduction

## Abstract

A variety of chiral *N*-phosphinyl α-imino esters have been synthesized for the first time from ketoesters and phosphinylamide, which were then reduced by L-selectride to give the corresponding *N*-phosphinyl-protected α-amino esters. The reduction proceeded very well with excellent chemical yields (88–98%) as well as high diastereoselectivities (96:4 to 99:1). Some of these products could be obtained without column chromatography and recrystallization. The chiral phosphinyl auxiliary could be easily cleaved under acidic conditions.

## Introduction

α-Imino esters play a very important role in the field of imine chemistry [1–3], because the ester group can serve as an activator to enhance the reactivity of the C=N double bond, thus making the following nucleophilic addition easier [4–5]. More attractively, the transformation of α-imino esters can provide an easy access to various natural and unnatural α-amino acid esters [6–10]. Up to now, a series of nucleophilic substrates have been reported to react with α-imino esters, such as enamine [11–14], carbamate ammonium ylide [15], 1,3-dipolar cycle [16], boronic acid [17], acetylide [18], proparygylic anion [19] and ketene silyl acetal [20]. These resulted α-amino acid derivatives are useful building blocks in modern organic synthetic and medicinal chemistry [21–22]. For example, we can easily find the active participation of α-imino esters in the total synthesis of the bioactive molecule fumimycin [23]. Moreover, α-amino acids are the most general fragments constituting peptides and proteins, especially for human insulin [24–28].

One of the most straightforward methods to prepare α-amino esters is the direct reduction of α-imino esters [29–30]. Several heterocyle-based reduction reagents have been developed for this transformation in the past years [31–35]. They usually served as mild hydrogen donors, but the use of them also made the purification difficult at the same time. Hydrogen gas is a traditional reduction source [36] and silanes and boranes are also well documented as the choices of reduction reagents [37–39].

**Figure 1:**
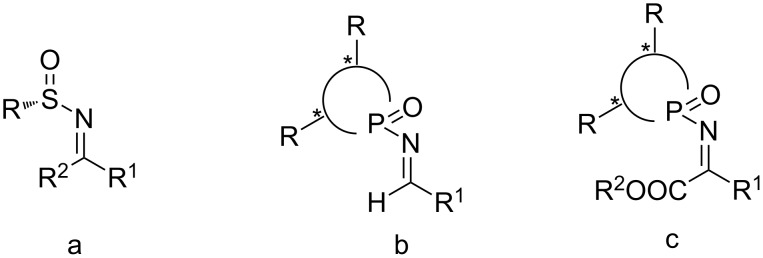
Typical sulfinyl (a), phosphonyl aldimines (b) and phosphinyl imino esters (c).

Recently, our groups have developed a new type of chiral phosphonyl auxiliaries for imines [40–44]. Compared with the reported sulfinyl auxiliaries (Figure 1a) [45–48], the phosphonyl ones exhibited advantages in the concise preparation method from readily available starting materials, an easy modification of the auxiliary and a highly diastereoselective control. Furthermore, the phosphonyl auxiliaries can do favor in the final purification, and it did not need any column chromatography or recrystallization, which was summarized as GAP (group assisted purification) [49–51]. So far, a number of *N*-phosphonyl aldimines (Figure 1b) have been synthesized and successfully used in many asymmetric additions [52–54]. In our continuous efforts on the chiral *N*-phosphonyl imine chemistry, we tried to develop novel *N*-phosphinyl protected α-imino esters and to use them for the asymmetric synthesis of α-amino esters. Herein, we report for the first time the synthesis of *N*-phosphinyl-protected α-imino esters (Figure 1c), followed by the reduction of these α-imino esters by L-selectride, to give the corresponding α-amino esters with excellent yields (88–98%) and virtually complete diastereoselectivities.

## Results and Discussion

### Preparation of the *N*-phosphinyl-protected α-imino esters

**Scheme 1:**
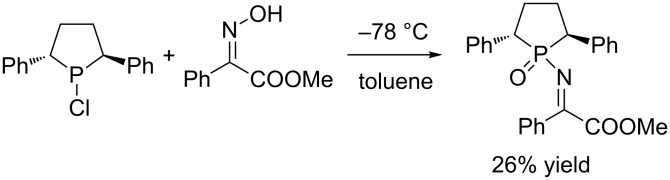
Synthesis of α-imino ester by rearrangement.

After screening the classical procedures for the synthesis of α-imino esters [3,55–56], we initially tried an approach with chlorophosphine and ester oxime as starting materials, which proceeded through rearrangement at low temperature (Scheme 1). ^31^P NMR and TLC of the crude product disclosed that several byproducts formed in this system. The isolation of the *N*-phosphinyl α-imino esters was very difficult and only 26% isolated yield of this product was obtained.

This unacceptable result shifted our attention to the previously reported methods for the preparation of *N*-phosphonyl aldimines, which was the condensation between aldehyde and phosphonyl amide [40–41]. Then, we carried out the optimization of the reaction conditions for the condensation of keto-ester **2a** and phosphinyl amide **1** (Table 1). Common dehydrating reagents, like 4 Å molecular sieves and magnesium sulfate, resulted in no desired product neither at room temperature nor under reflux (Table 1, entries 1–4). Very poor chemical yields of α-imino ester **3a** were detected when tetraethoxytitanium or tetraisopropoxytitanium was used as condensing reagent (Table 1, entries 5–8). Further treatment with titanium tetrachloride/triethyl amine gave a positive result and 46% chemical yield was obtained (Table 1, entry 9). Then, further investigation revealed that the best yield (47%) was observed when the reaction was conducted in the presence of TiCl_4_/Et_3_N at room temperature for 12 h (Table 1, entries 9–16). ^31^P NMR of the crude product predicted almost complete consumption of phosphinyl amide **1** but yields were very low. After careful study, we found that the obtained α-imino ester was not stable and decomposed slowly along with column chromatography purification and storage. For this reason, this α-imino ester was directly used in the following reaction after column chromatography to avoid decomposition.

**Table 1:** Optimization of the synthesis of α-imino ester **3a** by the condensation method.^a^

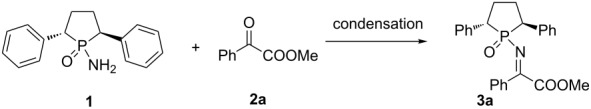					

entry	reagent	*T* (°C)	solvent	time (h)	yield (%)^b^

1	4 Å MS	rt	CH_2_Cl_2_	24	NR
2	4 Å MS	reflux	CH_2_Cl_2_	24	NR
3	MgSO_4_	rt	CH_2_Cl_2_	24	NR
4	MgSO_4_	reflux	CH_2_Cl_2_	24	NR
5	Ti(OEt)_4_/Et_3_N	rt	CH_2_Cl_2_	24	trace
6	Ti(OEt)_4_/Et_3_N	reflux	CH_2_Cl_2_	24	trace
7	Ti(OEt)_4_/Et_3_N	reflux	toluene	24	10
8	Ti(OiPr)_4_/Et_3_N	reflux	CH_2_Cl_2_	24	trace
9	TiCl_4_/Et_3_N	rt	CH_2_Cl_2_	24	46
10	TiCl_4_/Et_3_N	rt	CH_2_Cl_2_	12	47
11	TiCl_4_/Et_3_N	rt	CH_2_Cl_2_	6	33
12	TiCl_4_/Et_3_N	reflux	CH_2_Cl_2_	12	21
13	TiCl_4_/Et_3_N	rt	Et_2_O	12	20
14	TiCl_4_/Et_3_N	rt	THF	12	29
15	TiCl_4_/Et_3_N	rt	toluene	12	11
16	TiCl_4_/DIPEA	rt	CH_2_Cl_2_	12	31

^a^Rearrangement conditions: Phosphinyl amide **1** (0.5 mmol), ketoester **2a** (1.0 mmol), solvent (3.0 mL); ^b^isolated yields.

With the optimized conditions in hand, we then used varieties of keto-esters **2** as starting materials for the preparation of chiral α-imino esters **3** (Table 2). As shown in Table 2, modest yields were obtained (29–59%) for all the cases. In general, electron-deficient substrates performed better and the best yield was found for the substrate with *para*-fluoro substituent (Table 2, entry 4). The substrate with *ortho*-substituent led to lower yield due to steric effects (Table 2, entry 7). Besides, the reaction with ethyl ester also worked well and resulted in a slightly lower yield (Table 2, entry 11).

**Table 2:** Synthesis of *N*-phosphinyl-protected α-imino esters^a^.

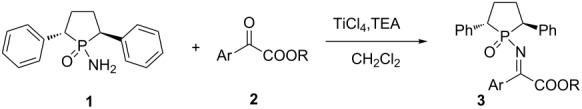				

entry	Ar	R	product	yield (%)^b^

1	C_6_H_5_	Me	**3a**	47
2	4-BrC_6_H_4_	Me	**3b**	50
3	4-ClC_6_H_4_	Me	**3c**	49
4	4-FC_6_H_4_	Me	**3d**	59
5	3-FC_6_H_4_	Me	**3e**	53
6	3-CH_3_C_6_H_4_	Me	**3f**	40
7	2-CH_3_C_6_H_4_	Me	**3g**	38
8	3,5-Cl_2_C_6_H_3_	Me	**3h**	29
9	2-Naphthyl	Me	**3i**	26
10	4-Ph-C_6_H_4_	Me	**3j**	35
11	C_6_H_5_	Et	**3k**	43

^a^Reaction conditions: Phosphinyl amide **1** (0.5 mmol), ketoester **2** (1.0 mmol), titanium(IV) chloride (0.5 mmol), triethylamine (2.0 mmol), dichloromethane (4.0 mL), rt for 12 h; ^b^isolated yields.

### Optimization of the asymmetric reduction reaction conditions

Then, the obtained chiral *N*-phosphinyl α-imino esters were used for the asymmetric synthesis of α-amino esters through reduction. The reduction condition scan was firstly focused on the examination of reductants. A number of reductants, including Hantzsch ester, silanes, organoaluminum and boranes were tested in the system. Unfortunately, silanes failed to trigger the reduction (Table 3, entries 2 and 3). Hantzsch ester and DIBAL were also not suitable for this reduction, and almost no desired product was obtained (Table 3, entries 1 and 5). Lithium triethylborohydride (Table 3, entry 4) and sodium borohydride (Table 3, entry 6) were decent choices, giving modest yields and very poor diastereoselectivity. Further screening of reductants found that L-Selectride and N-Selectride were good candidates, especially for L-Selectride, 92% yield and virtually complete controlled diastereoselectivity (99:1) were found (Table 3, entry 7). It did not matter at all when the reaction time was shortened to 8 h. However, the yield decreased when the time was further shortened to 6 h (Table 3, entries 9 and 10). The examination of solvents showed that polar solvents (Table 3, entries 11 and 13) were superior to a non-polar one such as toluene (Table 3, entry 12). The temperature influenced the reaction obviously, and raising the temperature to −40 °C reduces the yield to 89% and the diastereoselectivity to 87:13 (Table 3, entry 14).

**Table 3:** Optimization of asymmetric reduction of α-imino esters^a^.

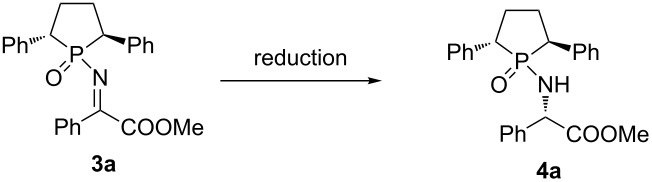						

entry	reagent	*T* (°C)	solvent	time (h)	Yield (%)^b^	dr^c^

1	Hantzsch ester	−78	THF	12	Trace	—
2	HSiCl_3_	−78	THF	12	NR	—
3	HSiEt_3_	−78	THF	12	NR	—
4	LiBHEt_3_	−78	THF	12	71	75:25
5	DIBAL	−78	THF	12	Trace	—
6	NaBH_4_	−78	THF	12	48	66:34
7	L-Selectride	−78	THF	12	92	99:1
8	N-Selectride	−78	THF	12	90	90:10
9	L-Selectride	−78	THF	8	92	99:1
10	L-Selectride	−78	THF	6	85	99:1
11	L-Selectride	−78	4-MeTHF	8	90	99:1
12	L-Selectride	−78	toluene	8	47	96:4
13	L-Selectride	−78	diethyl ether	8	82	99:1
14	L-Selectride	−40	THF	8	89	87:13

^a^Reaction conditions: α-imino ester **3a** (0.15 mmol), reduction reagent (0.30 mmol), solvent (5.0 mL); ^b^isolated yields; ^c^determined by ^31^P NMR of the crude reaction mixture.

### Scope of the asymmetric reduction reaction

After getting the optimized reduction conditions, several chiral *N*-phosphinyl α-imino esters prepared above were subjected to this system to examine the reaction scope. As shown in Table 4, reduction of these chiral α-imino esters provided excellent yields (up to 98%) and diastereoselectivities (up to 99:1). Almost all the reductions could achieve more than 90% yield, only for the case of *para*-phenyl-substituted substrate, a slightly lower yield was found (88%, Table 4, entry 10). The diastereoselectivities of the reactions were almost completely controlled, and most of them were 99:1. Except for the 4-bromo-substituted case, a dr of 96:4 was observed (Table 4, entry 2). It is interesting that GAP (group-assisted purification) was introduced into the reaction [49–51]. In five cases (Table 4, entries 1, 3–5 and 11), the final purified products were obtained by just washing with hexane, and no column chromatography or recrystallization was needed.

**Table 4:** Scope of novel phophinyl substituted α-amino esters.^a^

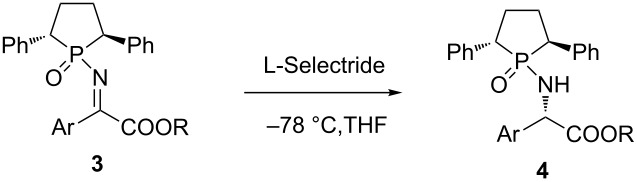						

entry	Ar	R	α-amino ester	purification method	yield (%)^b^	dr^c^

1	C_6_H_5_	Me	**4a**	GAP^d^	92	99:1
2	4-BrC_6_H_4_	Me	**4b**	Column	90	96:4
3	4-ClC_6_H_4_	Me	**4c**	GAP^d^	96	99:1
4	4-FC_6_H_4_	Me	**4d**	GAP^d^	98	99:1
5	3-FC_6_H_4_	Me	**4e**	GAP^d^	95	99:1
6	3-CH_3_C_6_H_4_	Me	**4f**	Column	95	99:1
7	2-CH_3_C_6_H_4_	Me	**4g**	Column	94	99:1
8	3,5-Cl_2_C_6_H_3_	Me	**4h**	Column	92	99:1
9	2-Naphthyl	Me	**4i**	Column	93	99:1
10	4-Ph-C_6_H_4_	Me	**4j**	Column	88	99:1
11	C_6_H_5_	Et	**4k**	GAP^d^	94	99:1

^a^Reaction conditions: α-imino esters **3** (0.15 mmol), L-Selectride (0.30 mmol), THF (5.0 mL); ^b^isolated yields; ^c^ determined by ^31^P NMR; ^d^group-assisted purification.

### Determination of the absolute configuration

**Scheme 2:**
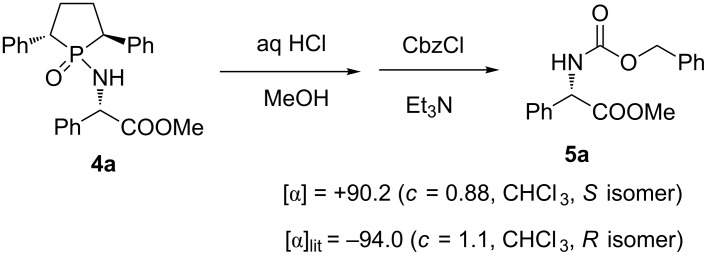
Cleavage of the chiral auxiliary.

The phosphinyl auxiliary could be easily cleaved by treating with concentrated hydrochloric acid in methanol. After stirring of **4a** with acid overnight, the conversion was complete and the corresponding amino ester hydrochloride was obtained. Then, the obtained free amino ester was directly converted into its *N*-Cbz-protected derivative **5a** by treating with CbzCl/Et_3_N (Scheme 2). The absolute configuration of the newly formed chiral center was assigned as *S* by comparing the optical rotation with that of known sample [57]. The stereochemical assignments of other products were made by analogy correspondingly.

## Conclusion

In conclusion, we have developed a method for the synthesis of chiral *N*-phophinyl α-imino esters for the first time, which have been used as precursors for asymmetric reductions with L-Selectride as reductant. Varieties of α-amino esters were obtained in excellent chemical yields and almost completely controlled diastereoselectivities. Furthermore, a part of the products could be purified without use of any column chromatography or recrystallization, which provides an alternative way for the synthesis of α-amino esters. The auxiliary was easily removed by treatment with acid to result the free α-amino esters.

## Experimental

**General procedure for the asymmetric reduction of *N*-phophinyl α-imino esters:** A reaction vial under argon was charged with L-Selectride (0.3 mmol) with THF (2.5 mL). The reaction mixture was then cooled to −78 °C for 10 min. Meanwhile, α-imino ester **3** (0.15 mmol, dissolved in 2.5 mL of THF) was cooled to −78 °C for 10 min. Then the α-imino ester **3** solution was transferred dropwise via a cannula at −78 °C and the reaction was kept at the same temperature for 8 h. The reaction mixture was quenched with saturated aqueous ammonium chloride (4.0 mL) and the organic layer was extracted with dichloromethane. The organic layers were dried over anhydrous Na_2_SO_4_, filtered and concentrated under reduced pressure. The crude product was purified using the GAP method or flash column chromatography on silica gel using EtOAc/hexanes (2:1, v/v) as the eluent to afford protected α-amino ester **4**.

## Supporting Information

File 1 Experimental details and spectral data.
