# Supplementary material for: Synthesis of chiral N-phosphinyl α-imino esters and their application in asymmetric synthesis of α-amino esters by reduction
Source: Beilstein J Org Chem. 2014 Mar 13;10:653–9. doi: 10.3762/bjoc.10.57 (PMC3999852; doi:10.3762/bjoc.10.57)

Supporting Information

for

Synthesis of chiral *N*-phosphinyl α-imino esters and their application in asymmetric synthesis of α-amino esters by reduction

Yiwen Xiong[1], Haibo Mei[1,2], Lingmin Wu[1], Jianlin Han*[1,2,3], Yi Pan*[1] and Guigen Li[2,4]

Address: [1]School of Chemistry and Chemical Engineering, State of Key Laboratory of Coordination, Nanjing University, Nanjing, 210093, China, [2]Institute for Chemistry & BioMedical Sciences, Nanjing University, Nanjing, 210093, China, [3]High-Tech Research Institute of Nanjing University, Changzhou, 213164, China and [4]Department of Chemistry and Biochemistry, Texas Tech University, Lubbock, Texas, 79409-1061, USA

Email: Jianlin Han* - hanjl@nju.edu.cn; Yi Pan* - yipan@nju.edu.cn

*Corresponding author

Experimental details and spectral data

Table of Contents:

1. **General information...S2**
2. **General procedure for synthesis of α-imino esters...........................S2**
3. **General procedure for asymmetric reduction of α-imino esters......S2**
4. **General procedure for cleavage of auxiliary....................................S3**
5. **Characterization data for 4 and 5...S4**
6. **^{1}H NMR and ^{13}C NMR spectra for compound 4 and 5..................S10**

1. General information

All the reactions were performed in oven-dried glassware and all commercially available reagents were used without further purification. Dichloromethane was distilled freshly from calcium hydride. Ether, toluene, THF, 2-MeTHF used for the reaction were distilled using benzophenone-sodium under nitrogen prior to use. Melting points are uncorrected. ^{1}H NMR, ^{13}C NMR spectra (TMS used as internal standard) and ^{31}P NMR spectra (85% phosphoric acid used as internal standard) was recorded at 400 MHz, 100 MHz and 162 MHz respectively in $CDCl_3$ with a Bruker ARX400 spectrometer. High resolution mass spectra for all new compounds were carried out by a Micro mass Q-Tof instrument (ESI). Optical rotation values were taken using an AUTOPOL IV automatic polarimeter. Analytical thin-layer chromatography (TLC) was performed by using glass-backed plates precoated with GF254 and the compounds were visualized with UV light (λ = 254 nm). Compounds were purified using flash column chromatography on silica gel 60 (200–300 mesh).

2. General procedure for synthesis of α-imino esters

Ketone ester **2** (1.0 mmol), phosphinyl amide **1** (0.5 mmol), triethylamine (1.0 mmol) in DCM (4.0 mL) were mixed and cooled in an ice bath, then titanium chloride (0.25 mmol in a 1 M DCM solution) was injected dropwise slowly during 30 min. After addition, the ice bath was removed and the solution was kept at rt 12 h. Then, the mixture was poured onto a pad of celite, washing the pad with 15 mL DCM. After concentration under reduced pressure, the residue was purified via column chromatography with ethyl acetate and petroleum ether (from 1:5 to 1:1 v/v) as eluent to give the product **3**.

3. General procedure for asymmetric reduction of α-imino esters

A reaction vial under argon was charged with L-selectride (0.3 mmol) with THF (2.5 mL). Reaction mixture was then cooled to −78 °C for 10 min. Meanwhile, α-imino ester **3** (0.15 mmol, dissolved in 2.5 mL of THF) was cooled to −78 °C for 10 min. Then α-imino ester **3** was transferred dropwise via a cannula at −78 °C and the

reaction was kept at the same temperature for 8 h. The reaction mixture was quenched with saturated aqueous ammonium chloride (4.0 mL) and the organic layer was extracted with dichloromethane. The organic layers were dried over anhydrous Na_2SO_4, filtered and concentrated under reduced pressure. The crude product was purified using GAP method or flash column chromatography on silica gel using EtOAc/hexanes (2:1, v/v) as the eluent to afford protected α-amino esters **4**.

4. General procedure for cleavage of auxiliary

N-Phosphinyl-protected α-amino ester **4a** (0.3 mmol) was dissolved in methanol (2.0 mL), concentrated hydrochloric acid (8.0 mL) was added dropwise during 5 min by syringe, and then the mixture was stirred at room temperature overnight. The solvent was removed in vacuo, and the residue was dissolved in DCM (5.0 mL), followed by addition of triethylamine (1.0 mmol). Then the solution was cooled on an ice bath before CbzCl (0.5 mmol) was added. The mixture was stirred at room temperature overnight until protection was complete then passed through celite. The celite was washed with DCM and the organic phases were combined. The crude product was purified using flash column chromatography on silica gel using EtOAc/hexanes (1:3 v/v) as the eluent to afford *N*-Cbz α-amino ester **5a**.

5. Characterization data for 4 and 5

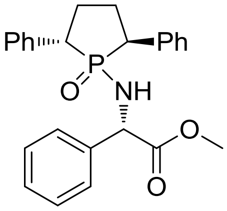

Compound 4a. White solid: mp 195-196 °C, $[\alpha]_D^{25}$ = -57.0 (c =0.91, $CHCl_3$); ^{1}H NMR (400 MHz, $CDCl_3$) δ 7.45-7.00 (m, 15H), 4.74 (dd, *J* = 10.0, 7.6 Hz, 1H), 3.77 (dd, *J* = 9.6, 8.0 Hz, 1H), 3.64-3.55 (m, 1H), 3.46 (s, 3H), 2.91-2.83 (m, 1H), 2.46-2.33 (m, 2H), 2.18-2.08 (m, 2H); ^{13}C NMR (100 MHz, $CDCl_3$) δ 172.0 (d, *J* = 6.3 Hz), 138.8 (d, *J* = 2.3 Hz), 136.6 (d, *J* = 4.9 Hz), 136.0 (d, *J* = 5.6 Hz), 129.1 (d, *J* = 2.3 Hz), 128.8 (d, *J* = 5.5 Hz), 128.7, 128.4 (d, *J* = 1.5 Hz), 128.1, 127.7 (d, *J* = 5.6 Hz), 127.2 (d, *J* = 2.7 Hz), 127.1, 126.7 (d, *J* = 2.2 Hz), 55.5, 52.7, 48.1, 47.3, 47.0, 46.1, 31.5 (d, *J* = 11.1 Hz), 27.1 (d, *J* = 10.3 Hz); ^{31}P NMR (162 MHz, $CDCl_3$) δ 52.6; HRMS-(ESI) *m/z* $[M+H]^+$ calcd for $C_{25}H_{27}NO_3P$, 420.1729; found, 420.1728.

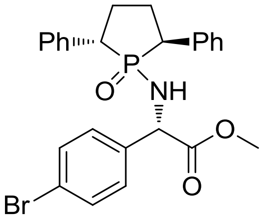

Compound 4b. White solid: mp 185-186 °C, $[\alpha]_D^{25}$ = -51.9 (c =1.12, $CHCl_3$); ^{1}H NMR (400 MHz, $CDCl_3$) δ 7.39-7.01 (m, 14H), 4.65 (dd, *J* = 10.4, 8.0 Hz, 1H), 3.91 (dd, *J* = 10.8, 8.0 Hz, 1H), 3.62-3.52 (m, 1H), 3.44 (s, 3H), 2.94-2.86 (m, 1H), 2.45-2.31 (m, 2H), 2.18-2.05 (m, 2H); ^{13}C NMR (100 MHz, $CDCl_3$) δ 171.6 (d, *J* = 6.6 Hz), 138.0 (d, *J* = 1.9 Hz), 136.3 (d, *J* = 4.9 Hz), 136.2 (d, *J* = 5.6 Hz), 131.7, 129.0 (d, *J* = 2.3 Hz), 128.8, 128.7, 128.66, 128.5 (d, *J* = 1.6 Hz), 127.7 (d, *J* = 4.6 Hz), 127.1 (d, *J* = 2.6 Hz), 127.1 (d, *J* = 2.6 Hz), 126.7 (d, *J* = 2.1 Hz), 122.1, 55.1, 52.7, 48.1, 47.4, 47.2, 46.4, 31.1 (d, *J* = 11.3 Hz),, 27.2 (d, *J* = 10.4 Hz); ^{31}P NMR (162 MHz, $CDCl_3$) δ 53.7; HRMS-(ESI) *m/z* $[M+H]^+$ calcd for $C_{25}H_{26}BrNO_3P$, 498.0834; found, 498.0831.

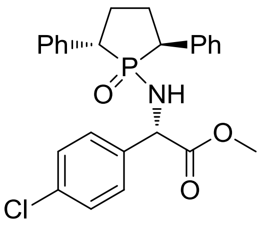

Compound 4c. White solid: mp 180-182 °C, $[\alpha]_D^{25}$ = -50.7 (c =0.92, $CHCl_3$); ^{1}H NMR (400 MHz, $CDCl_3$) δ 7.44-7.04 (m, 14H), 4.67 (dd, *J* = 10.0, 8.0 Hz, 1H), 3.76 (dd, *J* = 10.4, 7.6 Hz, 1H), 3.64-3.54 (m, 1H), 3.46 (s, 3H), 2.90-2.82 (m, 1H), 2.48-2.35 (m, 2H), 2.20-2.08 (m, 2H);\ ^{13}C NMR (101 MHz, $CDCl_3$) δ 171.7 (d, *J* = 6.6 Hz), 137.4 (d, *J* = 1.8 Hz), 136.3 (d, *J* = 5.0 Hz), 136.0 (d, *J* = 5.7 Hz), 134.0, 129.1 (d, *J* = 2.2 Hz), 128.7 (d, *J* = 5.5 Hz), 128.5, 128.4, 127.7 (d, *J* = 4.6 Hz), 127.2 (d, *J* = 2.6 Hz), 126.8 (d, *J* = 1.9 Hz), 55.0, 52.8, 48.0, 47.4, 47.3, 46.5, 31.3 (d, *J* = 11.3 Hz), 27.1 (d, *J* = 10.3 Hz); ^{31}P NMR (162 MHz, $CDCl_3$) δ 53.7; HRMS-(ESI) *m/z* $[M+H]^+$ calcd for $C_{25}H_{26}Cl\,NO_3P$, 454.1339; found, 454.1336.

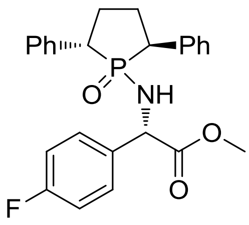

Compound 4d. White solid: mp 207-209 °C, $[\alpha]_D^{25}$ = -57.0 (c =0.88, $CHCl_3$); ^{1}H NMR (400 MHz, $CDCl_3$) δ 7.42-6.89 (m, 14H), 4.70 (dd, *J* = 10.0, 7.6 Hz, 1H), 3.84 (dd, *J* = 10.8, 8.0 Hz, 1H), 3.64-3.56 (m, 1H), 3.46 (s, 3H), 2.94-2.88 (m, 1H), 2.47-2.34 (m, 2H), 2.20-2.08 (m, 2H); ^{13}C NMR (100 MHz, $CDCl_3$) δ 171.5 (d, *J* = 6.3 Hz), 164.1, 161.6, 141.4 (d, *J* = 2.0 Hz), 141.3 (d, *J* = 2.0 Hz), 136.3 (d, *J* = 5.1 Hz), 136.0 (d, *J* = 5.6 Hz), 130.2 (d, *J* = 8.1 Hz), 129.1 (d, *J* = 2.3 Hz), 128.7 (d, *J* = 5.5 Hz), 128.5 (d, *J* = 1.6 Hz), 127.7 (d, *J* = 4.6 Hz), 127.1 (d, *J* = 2.7 Hz), 126.8 (d, *J* = 2.2 Hz), 122.7 (d, *J* = 2.8 Hz), 115.0 (d, *J* = 21.1 Hz), 114.0 (d, *J* = 22.4 Hz), 55.1 (d, *J* = 1.7 Hz), 52.8, 48.1, 47.3, 47.2, 46.4, 31.1 (d, *J* = 11.3 Hz), 27.1 (d, *J* = 10.4 Hz); ^{31}P NMR (162 MHz, $CDCl_3$) δ 53.5; HRMS-(ESI) *m/z* $[M+H]^+$ calcd for $C_{25}H_{26}FNO_3P$, 438.1634; found, 438.1634.

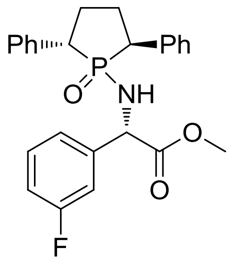

Compound 4e. White solid: mp 187-188 °C, $[\alpha]_D^{25}$ = -51.9 (c =1.03, $CHCl_3$); ^{1}H NMR (400 MHz, $CDCl_3$) δ 7.44- 6.90 (m, 14H), 4.69 (dd, J = 10.0, 7.6 Hz, 1H), 3.73 (dd, J = 10.0, 7.6 Hz, 1H), 3.64-3.54 (m, 1H), 3.47 (s, 3H), 2.90-2.82 (m, 1H), 2.49-2.34 (m, 2H), 2.19-2.12 (m, 2H); ^{13}C NMR (100 MHz, $CDCl_3$) δ 171.9 (d, J = 6.4 Hz), 163.7, 161.3, 139.3, 136.4 (d, J = 5.0 Hz), 136.0 (d, J = 5.7 Hz), 134.6 (d, J = 2.9 Hz), 129.1 (d, J = 2.3 Hz), 128.8 (d, J = 4.9 Hz), 128.7 (d, J = 2.2 Hz), 128.4 (d, J = 1.6 Hz), 127.7 (d, J = 4.5 Hz), 127.2 (d, J = 2.8 Hz), 126.8 (d, J = 2.2 Hz), 115.6 (d, J = 21.6 Hz), 54. 9, 52.7, 48.0, 47.3, 46.5, 31.4 (d, J = 11.2 Hz), 27.0 (d, J = 10.3 Hz); ^{31}P NMR (162 MHz, $CDCl_3$) δ 53.1; HRMS-(ESI) *m/z* $[M+H]^+$ calcd for $C_{25}H_{26}FNO_3P$, 438.1634; found, 438.1633

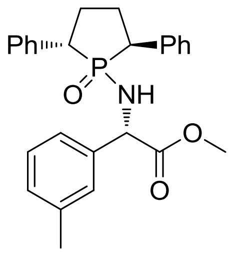

Compound 4f. White solid: mp 186-188 °C, $[\alpha]_D^{25}$ = -50.2 (c =0.73, $CHCl_3$); ^{1}H NMR (400 MHz, $CDCl_3$) δ 7.45-6.96 (m, 14H), 4.70 (dd, J = 9.6, 7.6 Hz, 1H), 3.72 (dd, J = 10.0, 7.6 Hz, 1H), 3.63-3.55 (m, 1H), 3.46 (s, 3H), 2.88-2.81 (m, 1H), 2.46-2.33 (m, 2H), 2.28 (s, 3H), 2.17-2.11 (m, 2H); ^{13}C NMR (100 MHz, $CDCl_3$) δ 170.9 (d, J = 6.5 Hz), 142.2, 139.3, 136.0 (d, J = 6.4 Hz), 135.7 (d, J = 5.3 Hz), 135.1, 129.1 (d, J = 2.3 Hz), 128.6, 128.5, 128.3, 127.6 (d, J = 4.7 Hz), 127.2 (d, J = 2.8 Hz), 127.0 (d, J = 2.2 Hz), 125.6, 114.1, 54.8, 53.0, 48.0, 47.8, 47.2, 47.0, 30.8 (d, J = 11.6 Hz), 27.2 (d, J = 10.5 Hz); ^{31}P NMR (162 MHz, $CDCl_3$) δ 52.5; HRMS-(ESI) *m/z* $[M+H]^+$ calcd for $C_{26}H_{29}FNO_3P$, 434.1885; found, 434.1885.

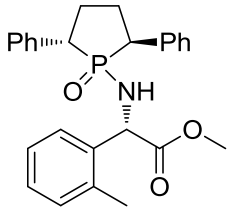

Compound 4g. White solid: mp 193-194 °C, $[\alpha]_D^{25}$ = -52.1 (c =0.98, $CHCl_3$); ^1H NMR (400 MHz, $CDCl_3$) δ 7.43, 7.41-7.02 (m, 14H), 4.70 (dd, J = 10.0, 7.6 Hz, 1H), 3.70 (dd, J = 8.4 Hz, 1H), 3.46 (s, 3H), 2.90-2.82 (m, 1H), 2.46-2.34 (m, 2H), 2.32 (s, 3H), 2.18-2.09 (m, 2H); ^{13}C NMR (100 MHz, $CDCl_3$) δ 172.2 (d, J = 6.5 Hz), 137.9, 136.8 (d, J = 4.8 Hz), 136.0, 135.8 (d, J = 2.3 Hz), 129.4, 129.1 (d, J = 2.2 Hz), 128.8 (d, J = 5.6 Hz), 128.4 (d, J = 1.6 Hz), 127.7 (d, J = 4.5 Hz), 127.2 (d, J = 2.6 Hz), 127.0, 126.6 (d, J = 2.1 Hz), 55.3, 52.6, 48.1, 47.3, 46.9, 46.1, 31.6 (d, J = 11.0 Hz), 27.1 (d, J = 10.5 Hz), 21.1; ^{31}P NMR (162 MHz, $CDCl_3$) δ 52.3; HRMS-(ESI) *m/z* $[M+H]^+$ calcd for $C_{26}H_{29}FNO_3P$, 434.1885; found, 434.1883.

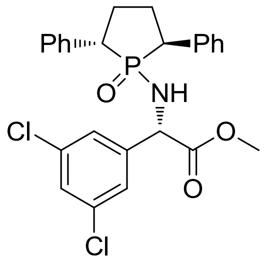

Compound 4h. White solid: mp 181-182 °C, $[\alpha]_D^{25}$ = -67.6 (c =1.01, $CHCl_3$); ^1H NMR (400 MHz, $CDCl_3$) δ 7.43- 7.05 (m, 13H), 4.60 (dd, J = 10.0, 7.6 Hz, 1H), 3.72 (dd, J = 11.1, 7.6 Hz, 1H), 3.65-3.55 (m, 1H), 3.49 (s, 3H), 2.94-2.86 (m, 1H), 2.51-2.39 (m, 2H), 2.23-2.08 (m, 2H); ^{13}C NMR (100 MHz, $CDCl_3$) δ 170.9 (d, J = 6.5 Hz), 142.3, 139.3, 136.0 (d, J = 5.6 Hz), 135.7 (d, J = 5.3 Hz), 135.1, 129.1 (d, J = 2.3 Hz), 128.6, 128.5, 128.3, 127.6 (d, J = 4.7 Hz), 127.2 (d, J = 2.8 Hz), 126.9 (d, J = 2.2 Hz), 125.6, 114.1, 54.8, 53.0, 48.0, 47.8, 47.2, 47.0, 30.8 (d, J = 11.6 Hz), 27.2 (d, J = 10.5 Hz); ^{31}P NMR (162 MHz, $CDCl_3$) δ 54.2; HRMS-(ESI) *m/z* $[M+H]^+$ calcd for $C_{25}H_{25}Cl_2NO_3P$, 488.0949; found, 488.0950.

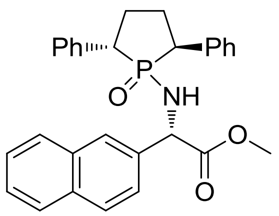

Compound 4i. White solid: mp 229-230 ^{o}C, $[\alpha]_D^{25}$ = -73.9 (c =1.04, $CHCl_3$); ^{1}H NMR (400 MHz, $CDCl_3$) δ 7.81- 6.90 (m, 17H), 4.89 (dd, J = 8.8 Hz, 1H), 3.86 (dd, J = 8.8 Hz, 1H), 3.62-3.55 (m, 1H), 3.47 (s, 3H), 2.85-2.80 (m, 1H), 2.46-2.32 (m, 2H), 2.13 (m, 2H); ^{13}C NMR (100 MHz, $CDCl_3$) δ 172.0 (d, J = 6.9 Hz), 136.4 (d, J = 4.9 Hz), 136.1, 136.0, 133.2 (d, J = 13.8 Hz), 129.1 (d, J = 2.1 Hz), 128.7, 128.6, 128.2, 128.1, 127.7 (d, J = 4.5 Hz), 127.6, 127.2, 126.7, 126.6, 126.3, 124.4, 55.8, 52.7, 48.1, 47.4, 47.1, 46.3, 31.6 (d, J = 11.1 Hz), 27.1 (d, J = 10.2 Hz); ^{31}P NMR (162 MHz, $CDCl_3$) δ 52.9; HRMS-(ESI) m/z $[M+H]^+$ calcd for $C_{29}H_{29}NO_3P$, 470.1885; found, 470.1886.

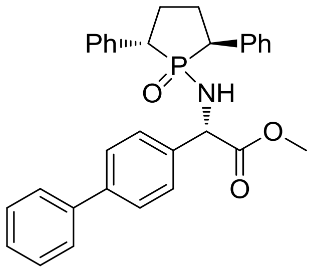

Compound 4j. White solid: mp 216-218 ^{o}C, $[\alpha]_D^{25}$ = -78.4 (c =1.18, $CHCl_3$); ^{1}H NMR (400 MHz, $CDCl_3$) δ 7.57-7.04 (m, 19H), 4.77 (dd, J = 10.0, 7.6 Hz, 1H), 3.75 (dd, J = 9.6, 7.6 Hz, 1H), 3.66-3.56 (m, 1H), 3.50 (s, 3H), 2.92-2.86 (m, 1H), 2.49- 2.36 (m, 2H), 2.20-2.12 (m, 2H); ^{13}C NMR (100 MHz, $CDCl_3$) δ 172.0 (d, J = 6.6 Hz), 141.0, 140.6, 137.7 (d, J = 2.1 Hz), 136.5 (d, J = 4.9 Hz), 136.0 (d, J = 5.7 Hz), 129.1 (d, J = 2.2 Hz), 128.8, 128.7, 128.4 (d, J = 1.6 Hz), 127.7 (d, J = 4.6 Hz), 127.5, 127.4, 127.2 (d, J = 2.7 Hz), 127.1, 126.7, 55.4, 52.7, 48.1, 47.3, 47.2, 46.4, 31.6 (d, J = 11.1 Hz), 27.1 (d, J = 10.3 Hz); ^{31}P NMR (162 MHz, $CDCl_3$) δ 52.8; HRMS-(ESI) m/z $[M+H]^+$ calcd for $C_{31}H_{31}NO_3P$, 496.2042; found, 496.2042.

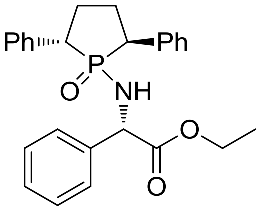

Compound 4k. White solid: mp 200-202 ^{o}C, $[\alpha]_D^{25}$ = -59.6 (c =0.97, $CHCl_3$); ^{1}H NMR (400 MHz, $CDCl_3$) δ 7.43-6.99 (m, 15H), 4.73 (dd, J = 10.0, 7.6 Hz, 1H), 3.96-3.88 (m, 2H), 3.73 (dd, J = 8.4 Hz, 1H), 3.61-3.55 (m, 1H), 2.89-2.82 (m, 1H), 2.45-2.34 (m, 2H), 2.18-2.11 (m, 2H), 1.04 (t, J = 7.6 Hz, 3H); ^{13}C NMR (100 MHz, $CDCl_3$) δ 171.4 (d, J = 6.4 Hz), 139.3, 138.9, 136.6 (d, J = 4.9 Hz), 136.0 (d, J = 5.8 Hz), 129.1, 128.8 (d, J = 5.6 Hz), 128.6, 128.4 (d, J = 1.6 Hz), 128.1, 127.7 (d, J = 4.5 Hz), 127.2 (d, J = 2.6 Hz), 114.1, 61.8, 55.6, 48.1, 47.3, 46.9, 46.1, 31.6 (d, J = 11.0 Hz), 27.1 (d, J = 10.2 Hz), 13.8; ^{31}P NMR (162 MHz, $CDCl_3$) δ 52.3; HRMS-(ESI) m/z $[M+H]^+$ calcd for $C_{26}H_{29}FNO_3P$, 434.1885; found, 434.1884.

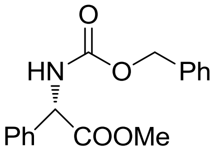

Compound 5a. Colorless oil: $[\alpha]_D^{25}$ = +90.2(c=0.88, $CHCl_3$); ^{1}H NMR (400 MHz, $CDCl_3$) δ 7.32-7.26 (m, 10H), 6.07 (d, J = 1.8 Hz, 1H), 5.38 (d, J = 1.8 Hz, 1H), 5.05 (q, J = 1.8 Hz, 2H), 3.62 (s, 3H) ; ^{13}C NMR (100 MHz, $CDCl_3$) δ 171.4, 155.5, 136.7, 136.3, 129.0, 128.6, 128.5, 128.2, 127.3, 67.1, 58.1, 52.8.

6. ^{1}H NMR and ^{13}C NMR spectra for compound 4 and 5 ($CDCl_3$)

The peaks in the high field (δ = 1.25) in the ^{1}H NMR spectra for the compounds **4b**–**4k**, are the peaks from solvents (hexanes or petroleum ether).

4a

^{1}H NMR

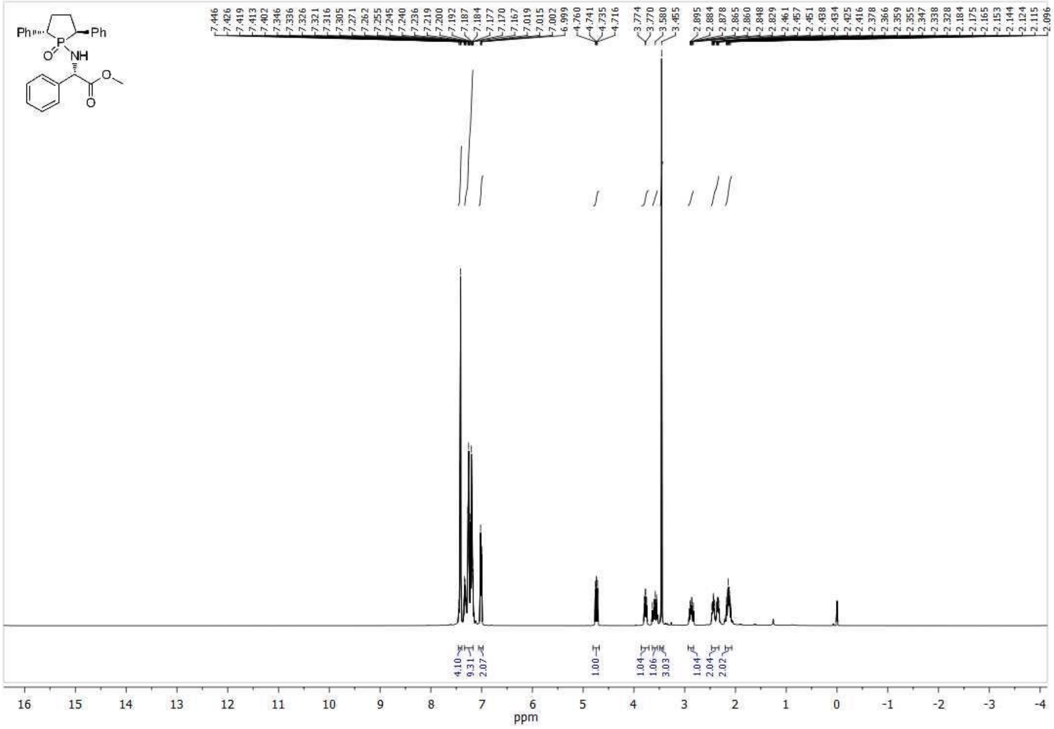

^{13}C NMR

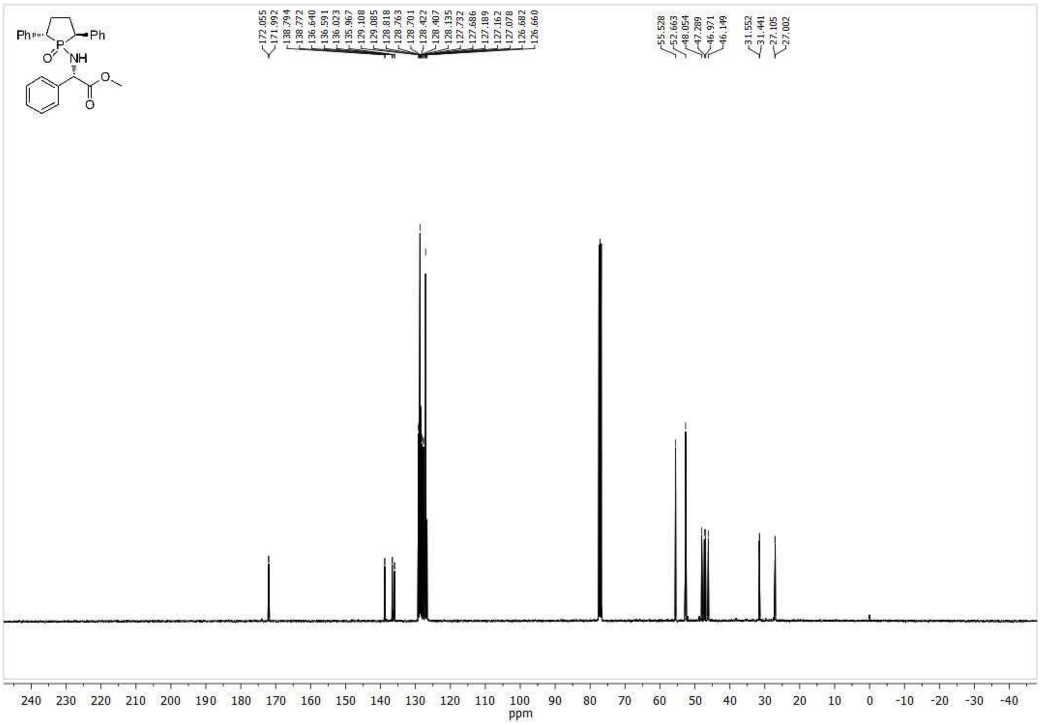

4b

^{1}HNMR

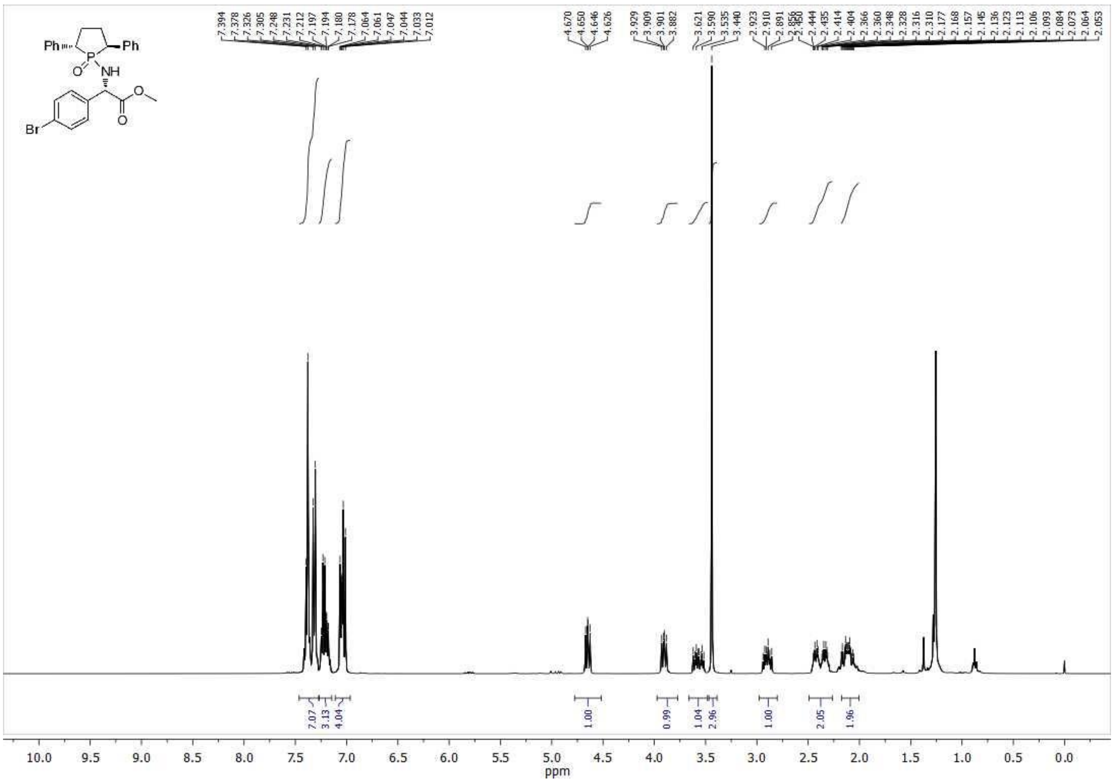

^{13}CNMR

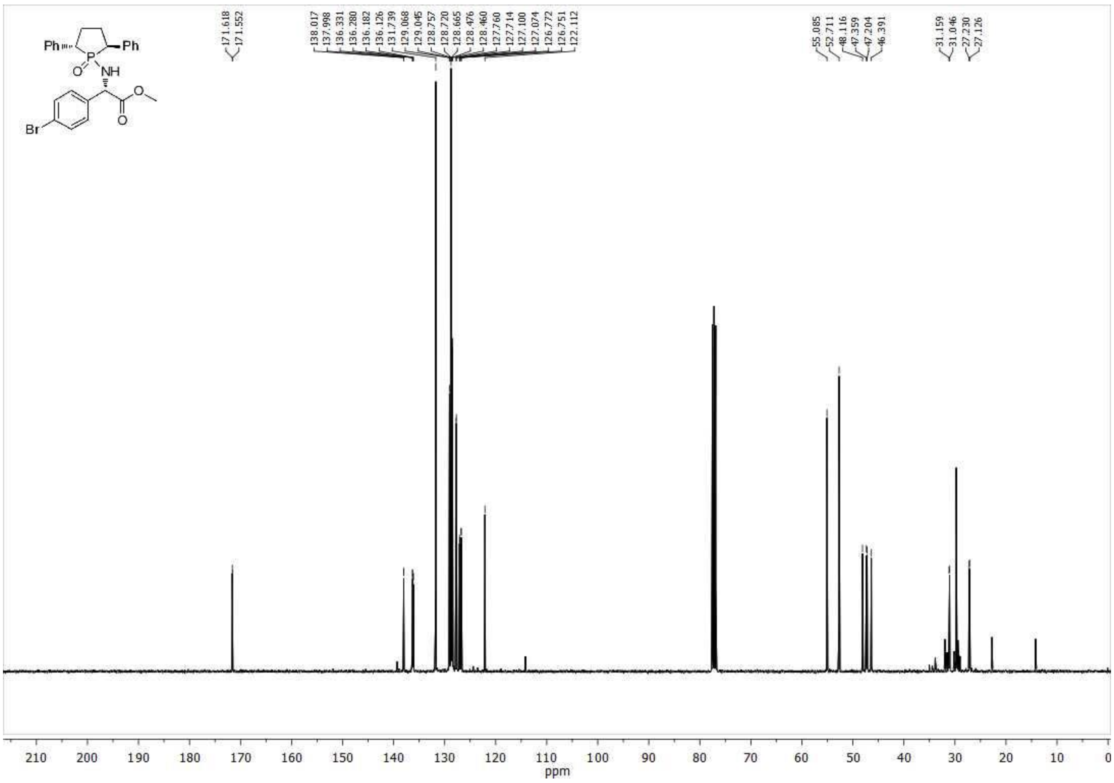

4c

^{1}HNMR

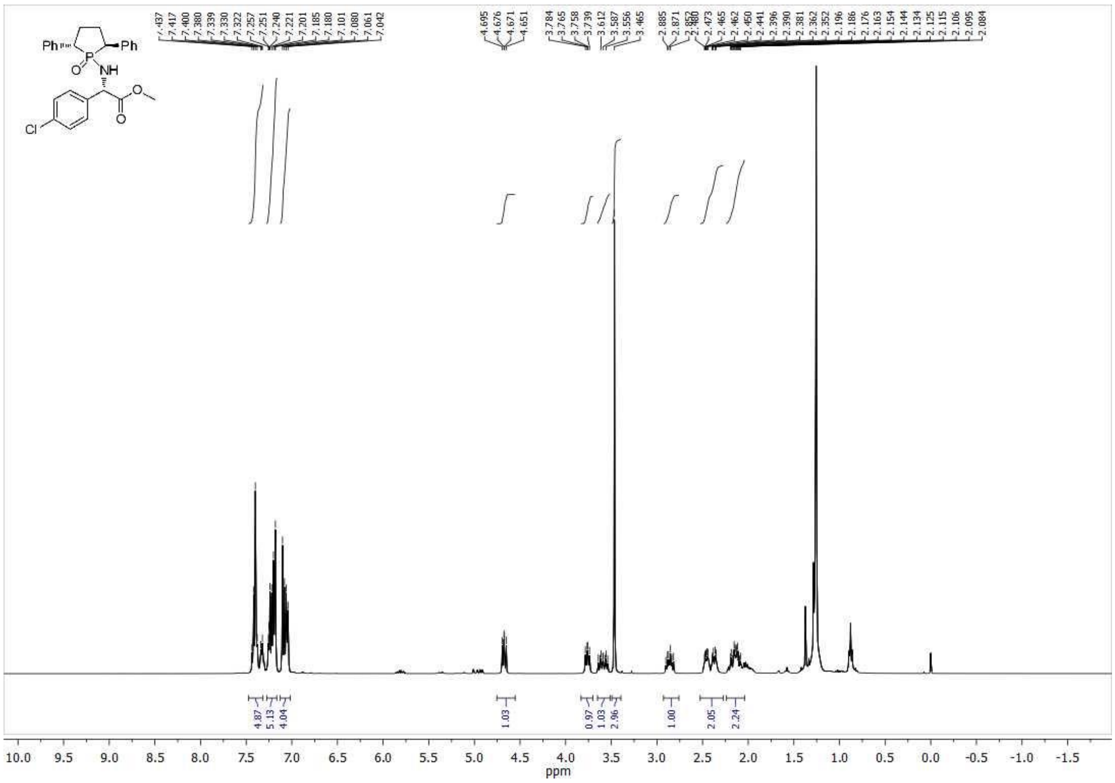

^{13}CNMR

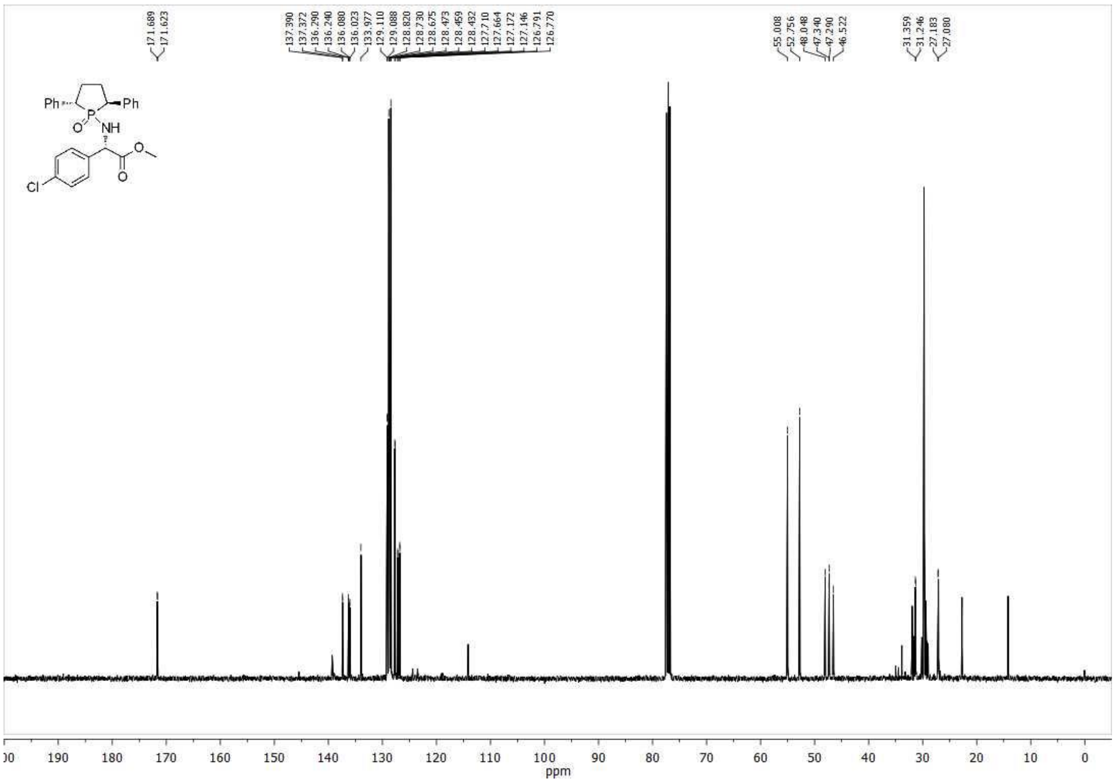

4d

^{1}HNMR

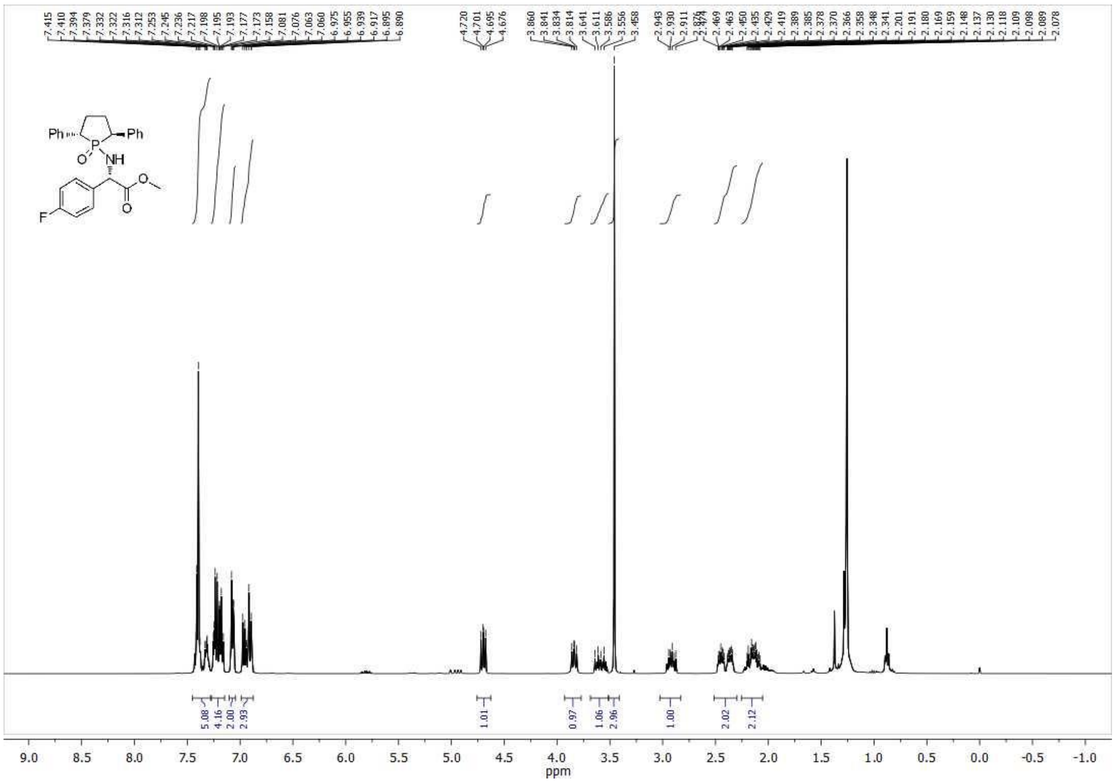

^{13}CNMR

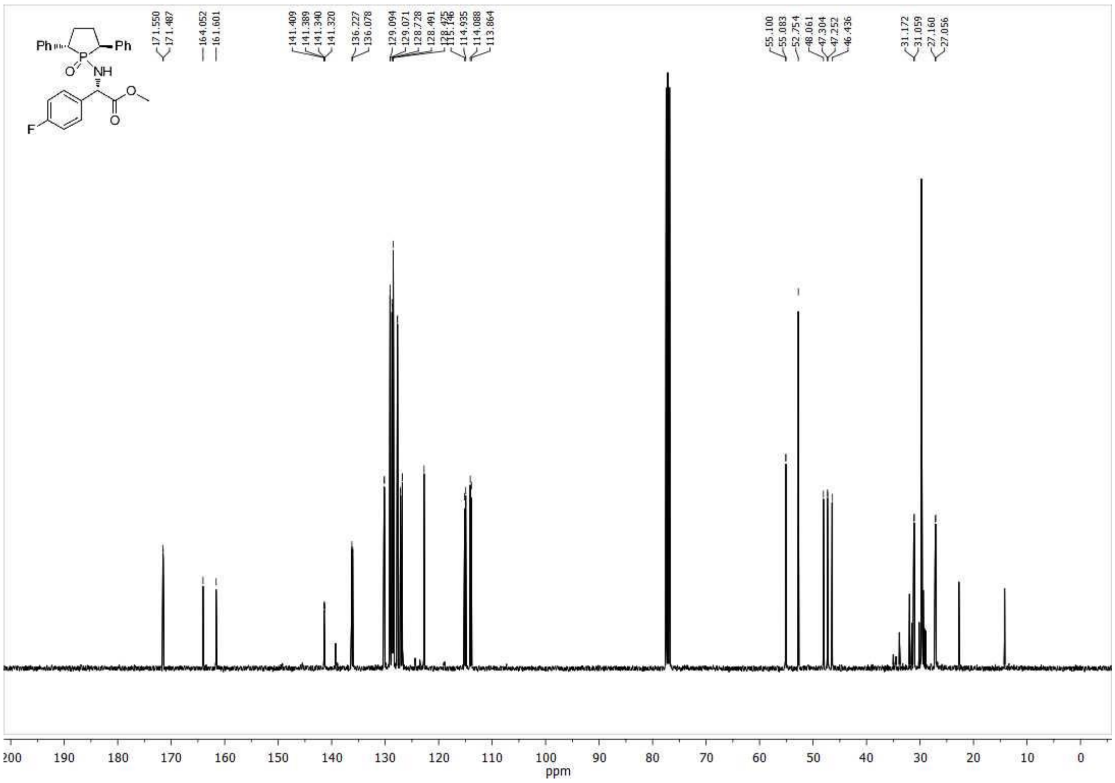

4e

^{1}HNMR

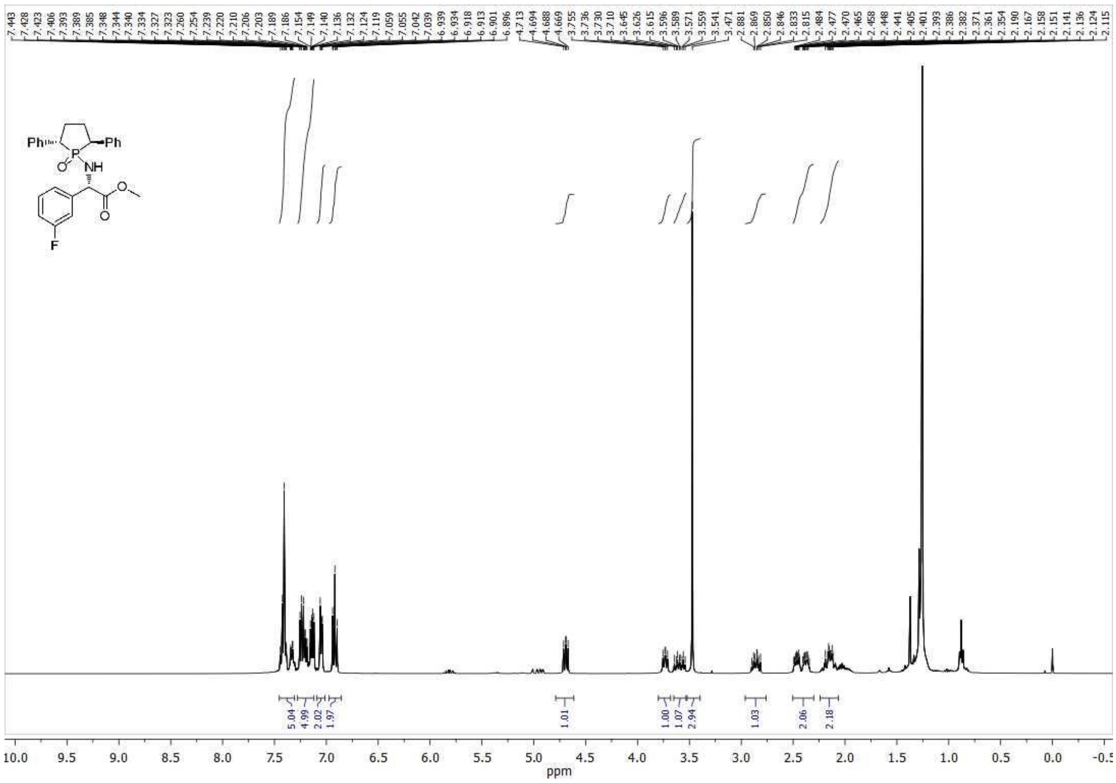

^{13}CNMR

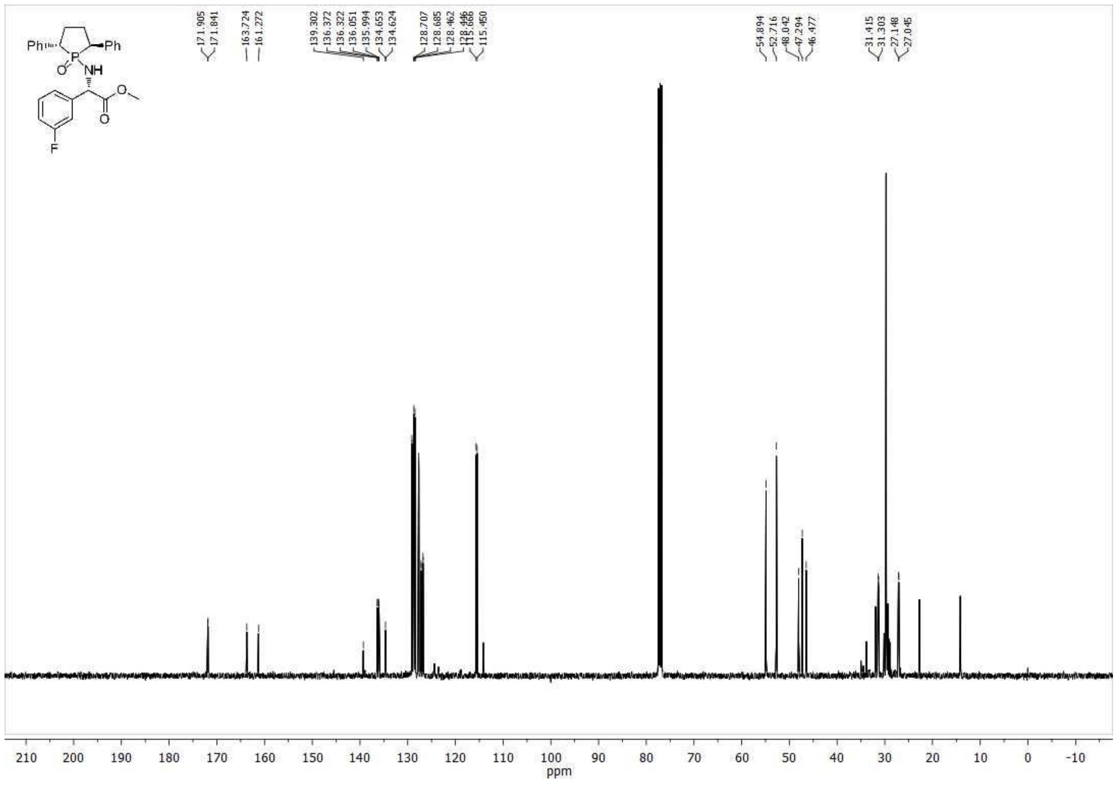

4f

^{1}HNMR

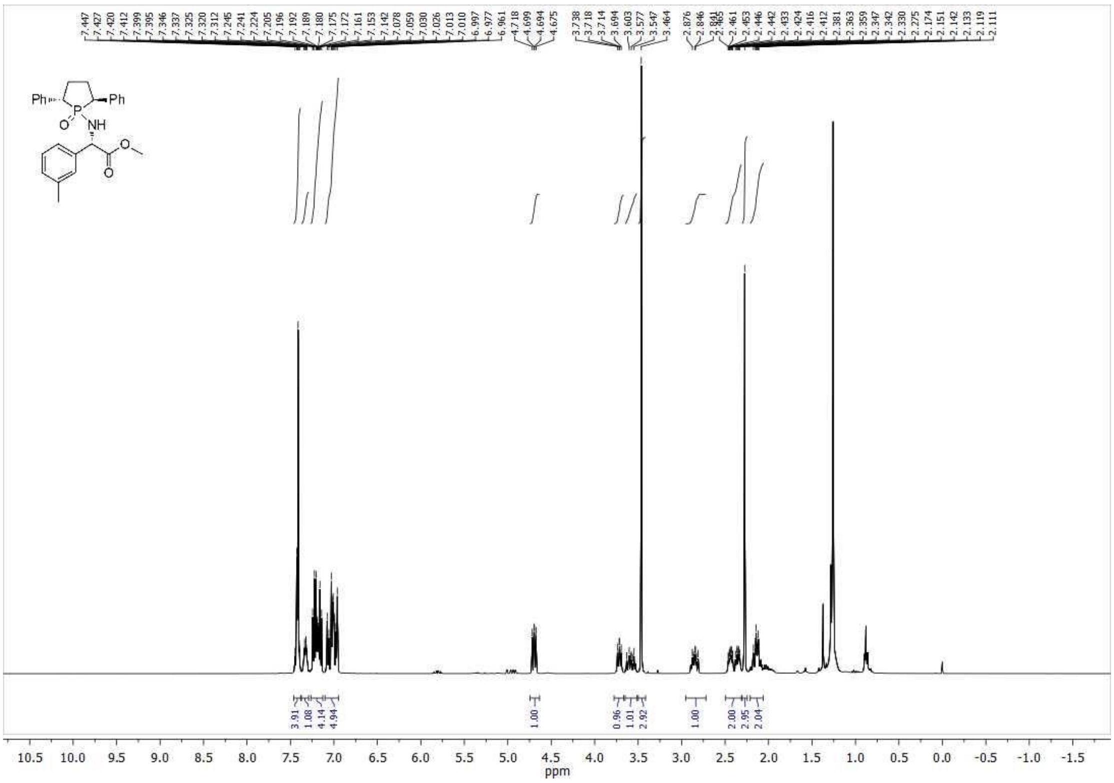

^{13}CNMR

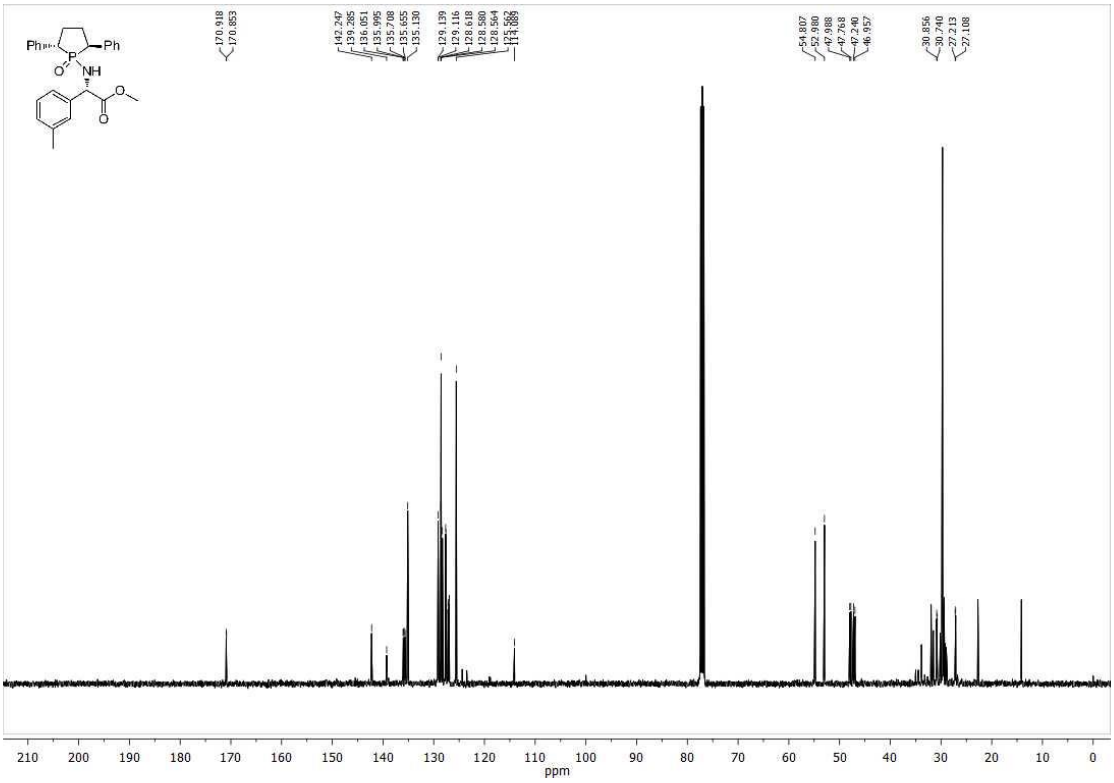

4g

^{1}HNMR

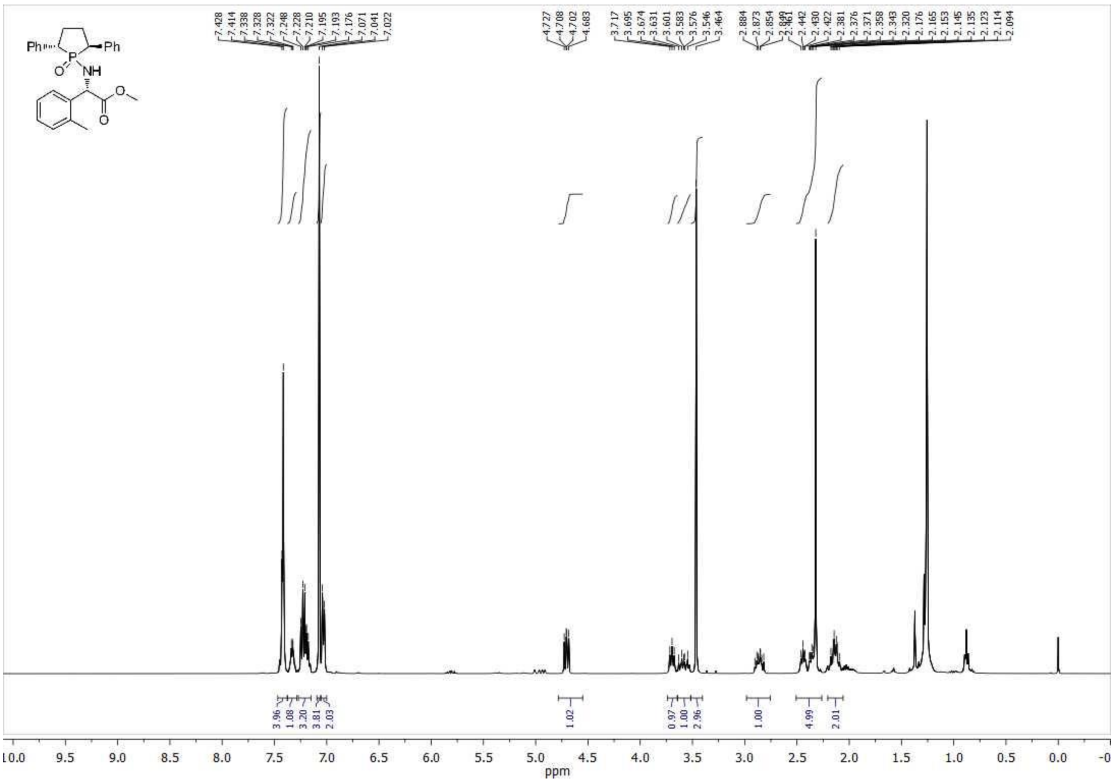

^{13}CNMR

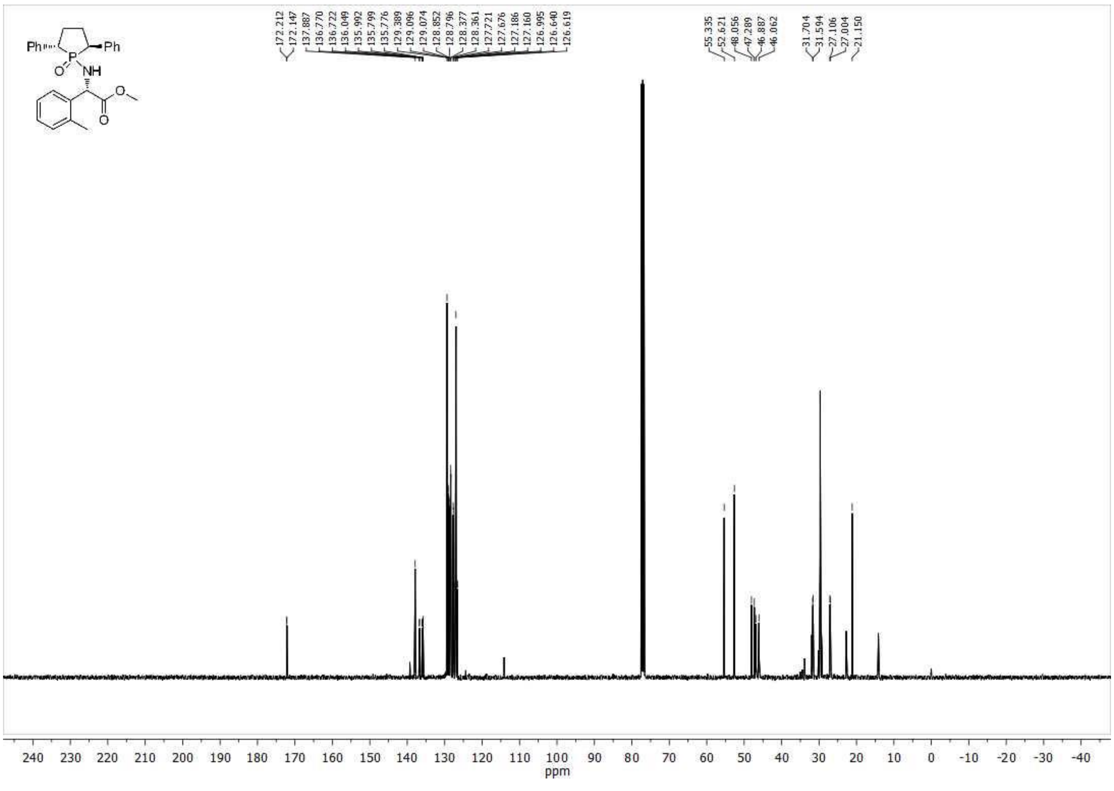

4h

^{1}HNMR

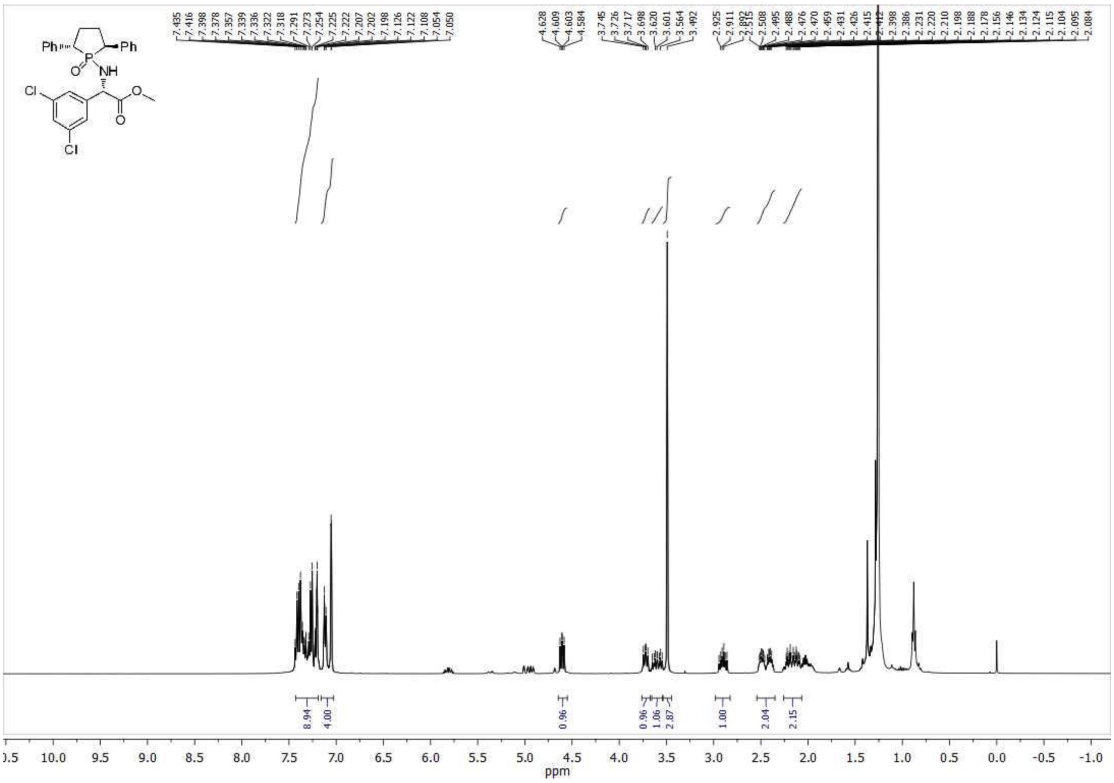

^{13}CNMR

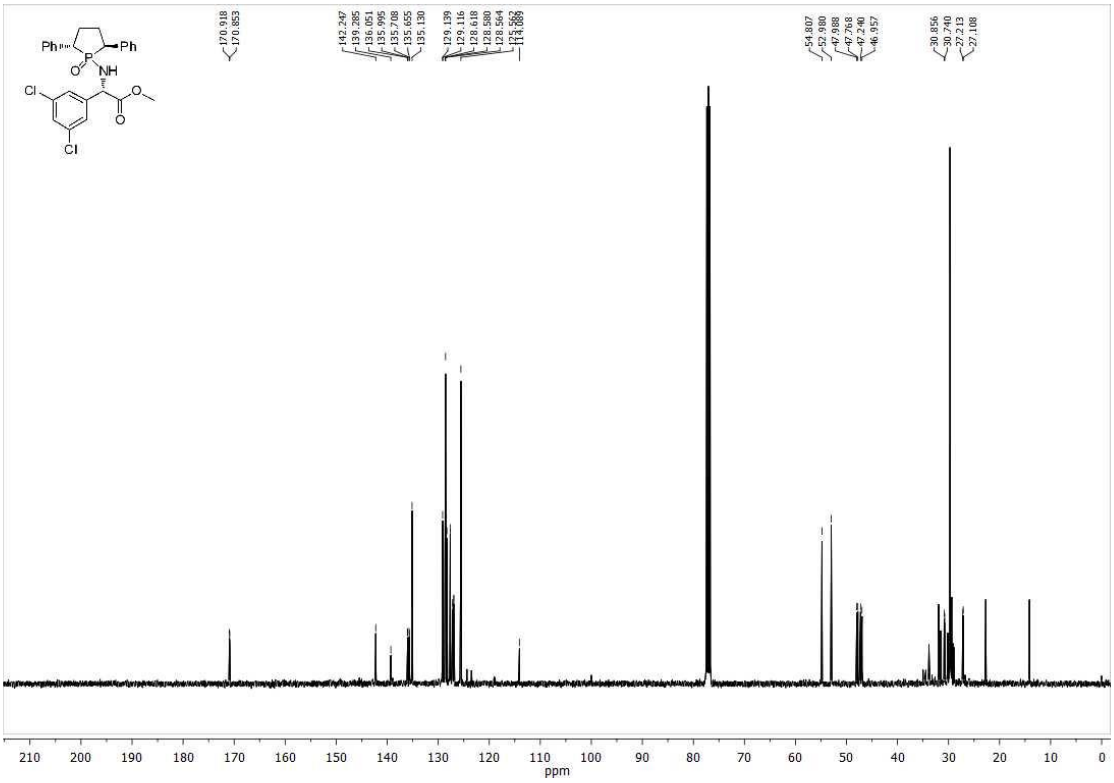

4i

^{1}HNMR

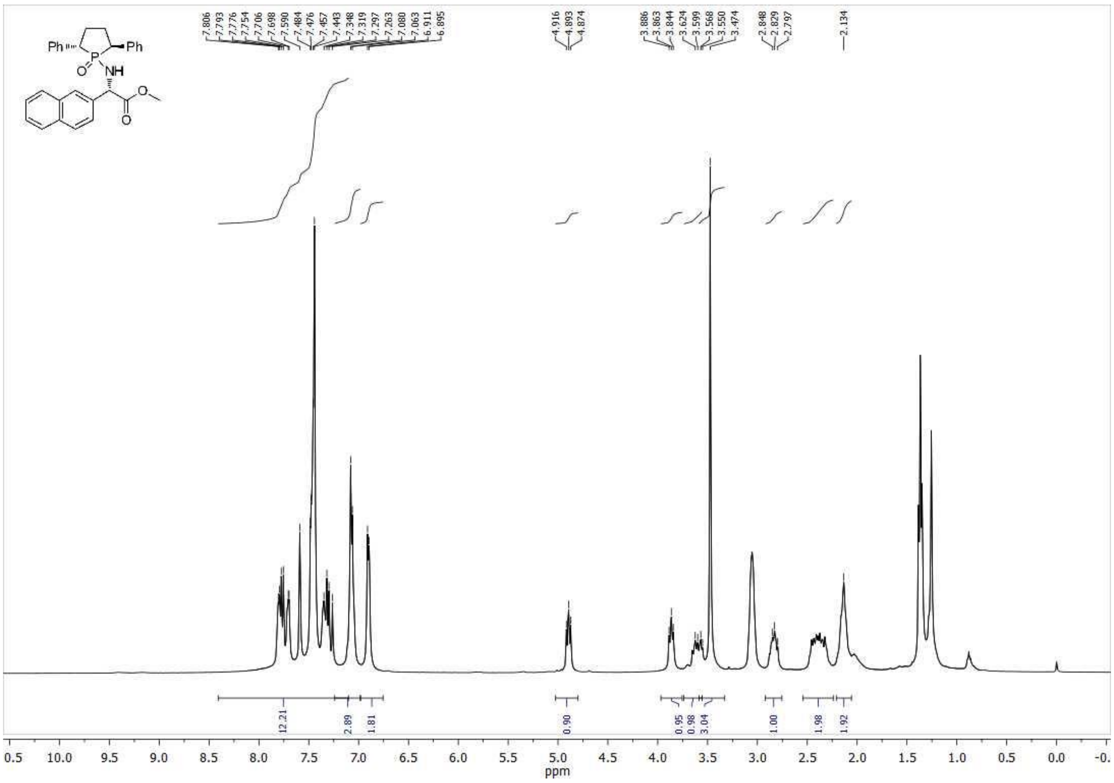

[31]CNMR

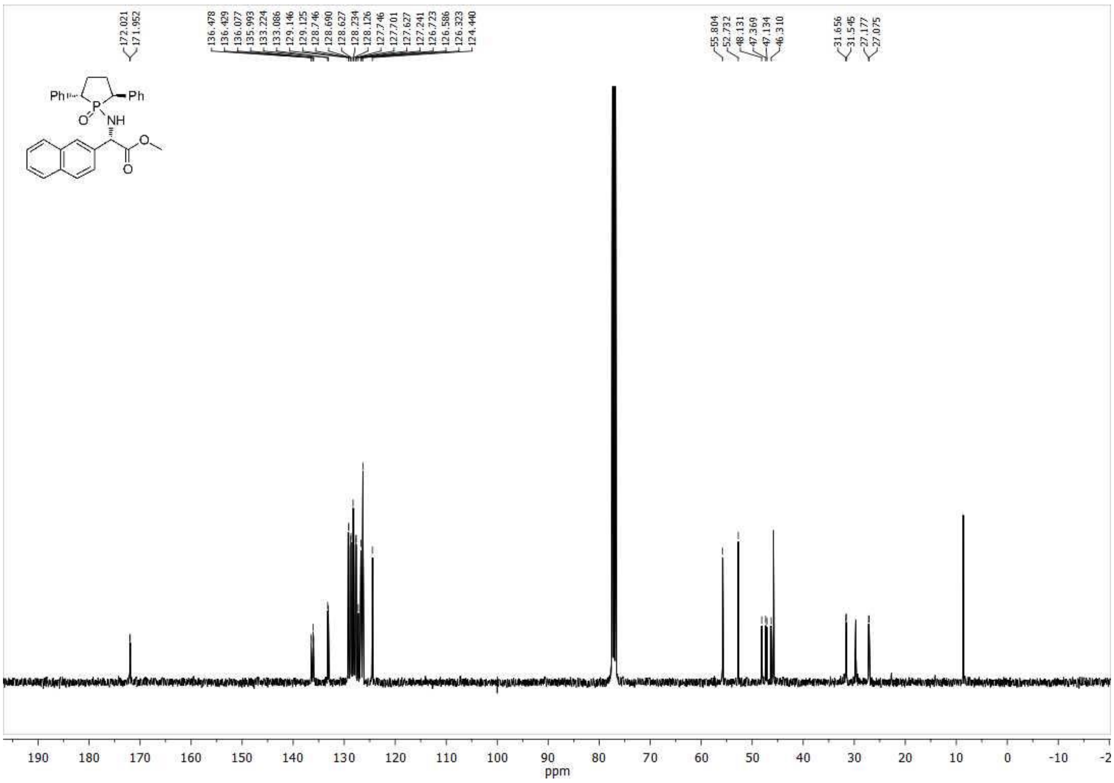

4j

^{1}HNMR

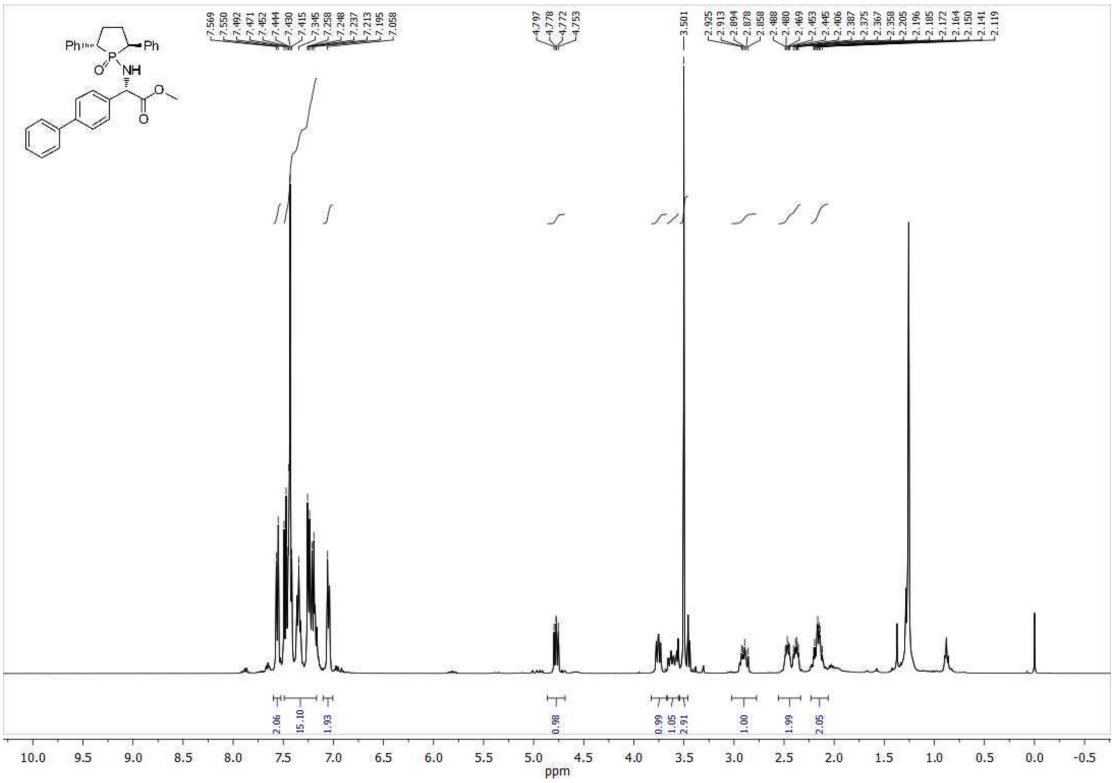

^{13}CNMR

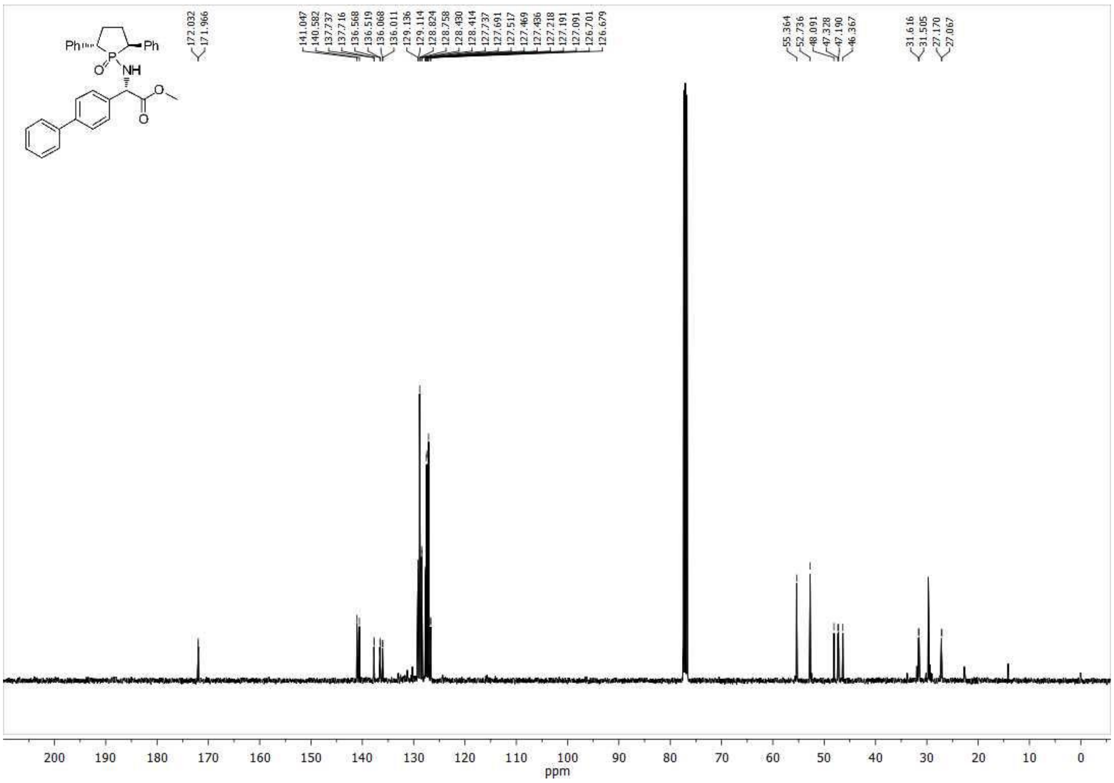

4k

^{1}HNMR

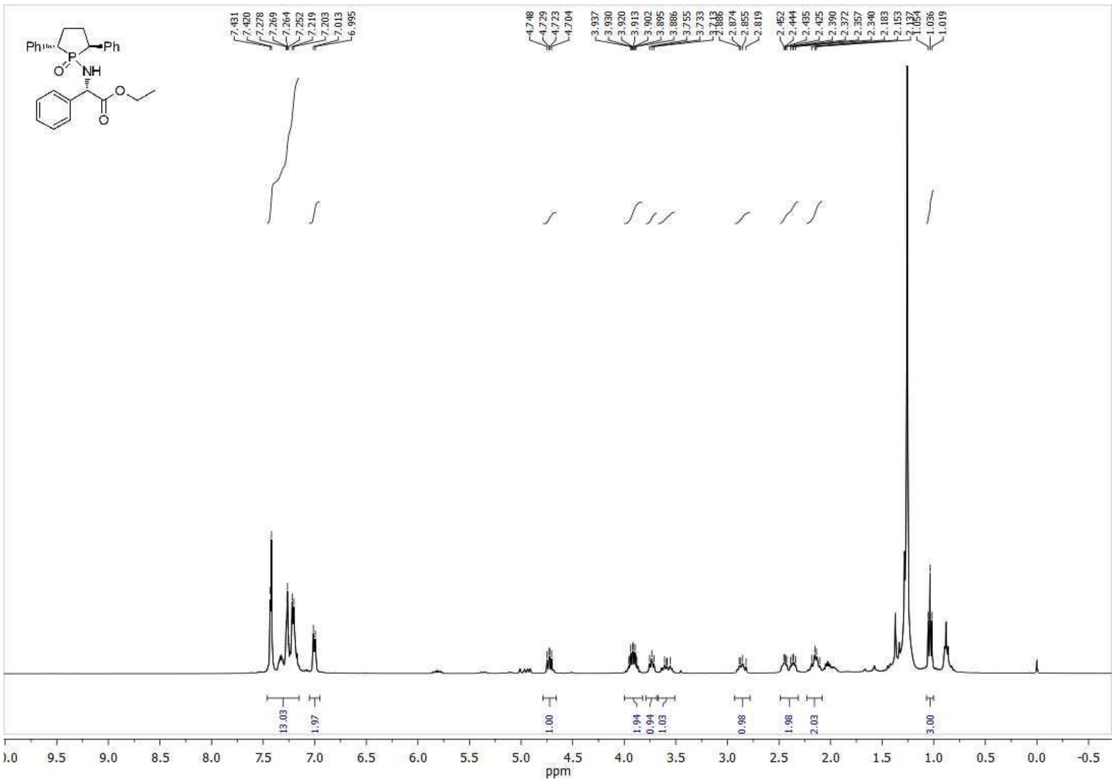

[31]CNMR

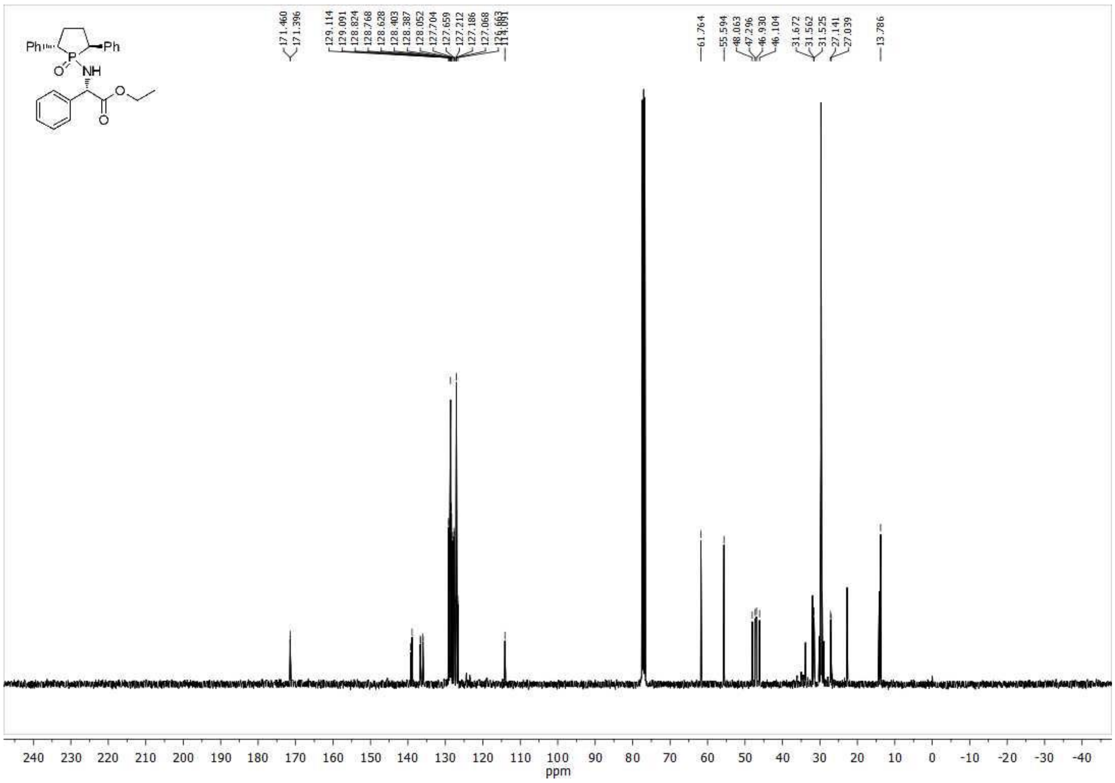

5a

^{1}HNMR

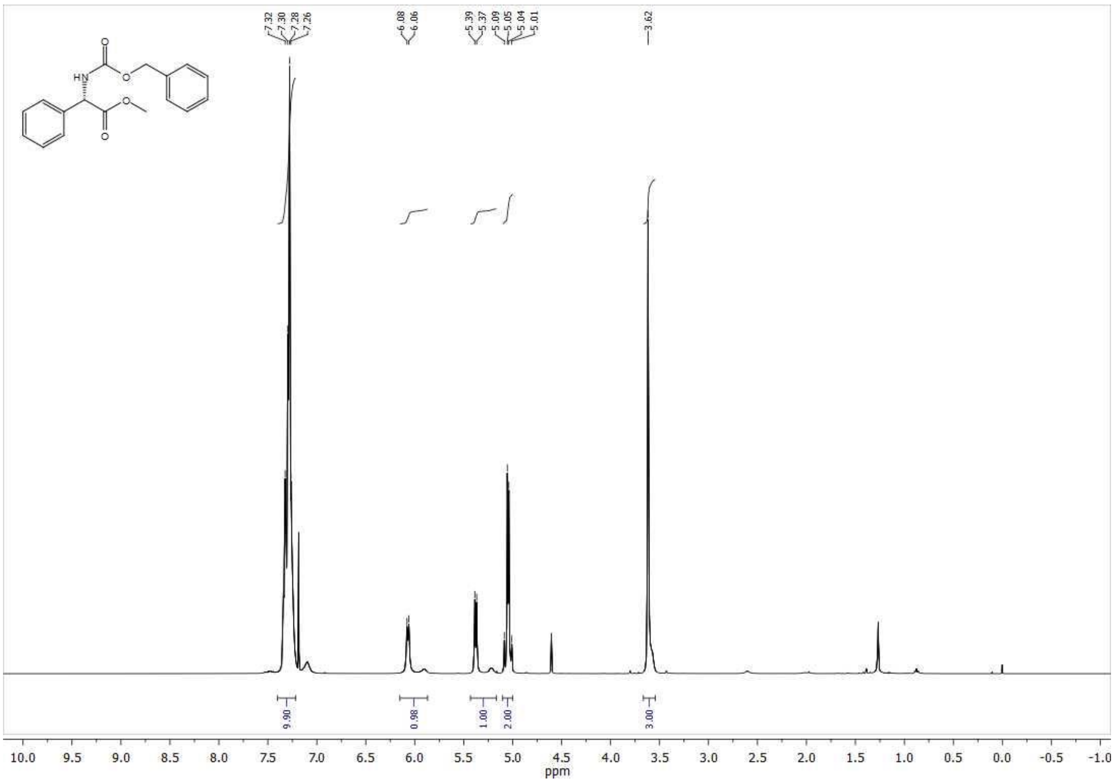

^{13}CNMR

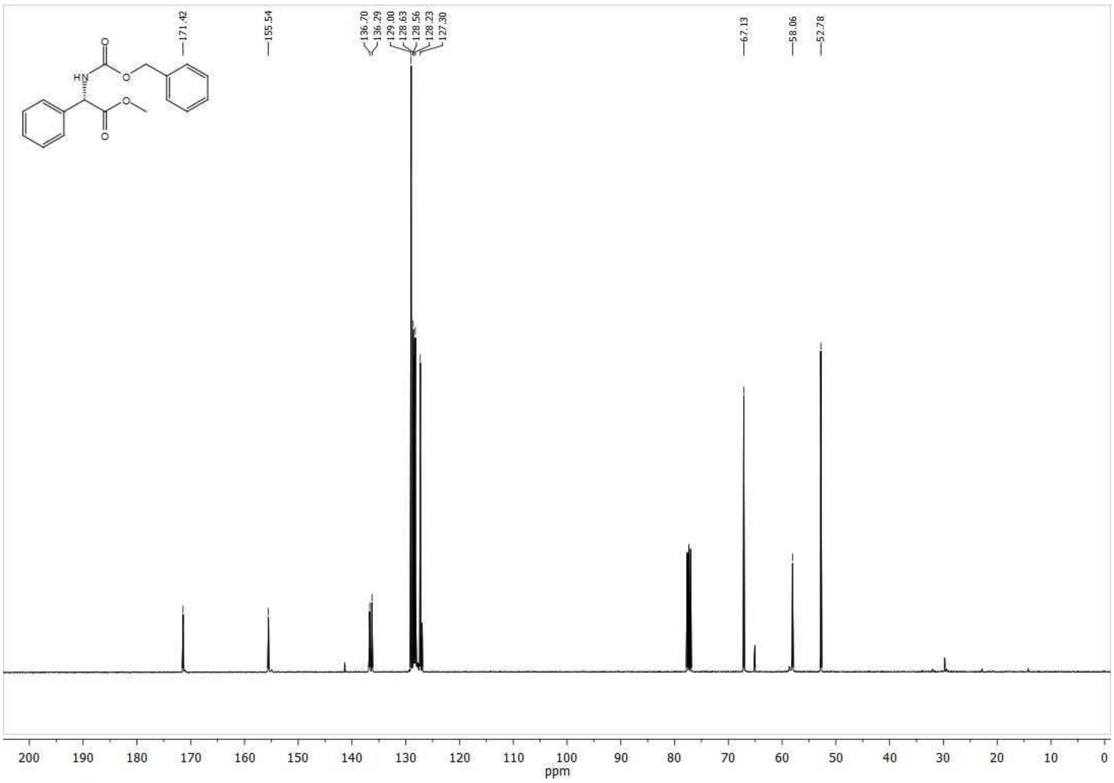

Supplement: File 1 — Experimental details and spectral data. [file Beilstein_J_Org_Chem-10-653-s001.pdf]
